# Decreased soil multifunctionality is associated with altered microbial network properties under precipitation reduction in a semiarid grassland

**DOI:** 10.1002/imt2.106

**Published:** 2023-05-02

**Authors:** Xing Wang, Qi Zhang, Zhenjiao Zhang, Wenjie Li, Weichao Liu, Naijia Xiao, Hanyu Liu, Leyin Wang, Zhenxia Li, Jing Ma, Quanyong Liu, Chengjie Ren, Gaihe Yang, Zekun Zhong, Xinhui Han

**Affiliations:** ^1^ College of Agronomy Northwest A&F University Yangling China; ^2^ Shaanxi Engineering Research Center of Circular Agriculture Yangling China; ^3^ Institute for Environmental Genomics and Department of Microbiology and Plant Biology University of Oklahoma Norman Oklahoma USA; ^4^ Institute of Soil and Water Conservation Northwest A&F University Yangling China

## Abstract

Our results reveal different responses of soil multifunctionality to increased and decreased precipitation. By linking microbial network properties to soil functions, we also show that network complexity and potentially competitive interactions are key drivers of soil multifunctionality.

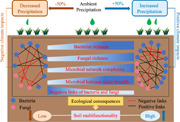

Global climate warming during the past few decades has resulted in the intensification of the hydrological cycle [1, 2], thereby leading to shifts in precipitation patterns at the global as well as regional scales [3, 4]. Such altered precipitation regimes are expected to have dramatic ecological consequences, including changes in microbial assembly and variations in biodiversity and ecosystem functions, especially in water‐constrained areas, such as arid and semiarid ecosystems [5, 6, 7]. Accounting for more than 40% of the earth's terrestrial surface [8], dryland ecosystems are highly sensitive to altered precipitation regimes due to their persistent low precipitation inputs [6, 9]. A substantial body of literature has documented how dryland ecosystems respond to increased and decreased precipitation; however, most studies have largely focused on the response of above‐ground plant communities [10, 11, 12, 13, 14]. Plant communities generally respond to altered precipitation in terms of productivity [15], phenology [16], community composition [17], and resource use [18], although the response is context‐dependent. By comparison, our understanding of the implications of altered precipitation regimes regarding below‐ground microbial communities in drylands and related soil functional dynamics is limited, even though this situation has improved in recent years [18, 19, 20, 21, 22]. A recent observational survey over large spatial scales underscored the importance of investigating the microbial responses to climate change in dryland ecosystems as they show that soil microbial diversity is a better predictor of the ecosystem function in relatively more arid regions compared to plant diversity [23]. In addition, the predicted rapid changes in precipitation regimes may pose a challenge for organisms (e.g., by inducing water or oxygen deficiency) [11, 24], while altering the biological interactions [25, 26]. Such effects may be more pronounced in already vulnerable, water‐limited dryland ecosystems, where the stability of biological relationships depends greatly on water availability [27, 28, 29]. In such cases, altered precipitation may reshape the microbial coexistence patterns, thereby causing a strong cascade effect on microbial‐mediated soil functions [6, 26, 30, 31]. Thus, conducting field studies in arid ecosystems to explore the general responses of microbial community structures to altered precipitation and the underlying mechanisms associated with ecosystem function is imperative.

Ecosystem function is inherently multifunctional, reflecting the ability of an ecosystem to deliver multiple functions or services simultaneously, such as water and fertilizer availability, elemental cycling, and organic matter decomposition [23, 31]. Numerous recent studies have shown that biodiversity—plant or microbial—supports ecosystem multifunctionality at the microcosm, regional, and global scales [23, 32, 33, 34]. For instance, a global survey reported a significant positive correlation between soil microbial diversity and ecosystem multifunctionality in drylands [35, 36]. However, there is a paucity of information on the effects of climate change (e.g., altered precipitation) on ecosystem multifunctionality and its relationship with microbial biodiversity. More importantly, the soil microbiome is highly structured [37, 38]; complex interactions, such as competition or symbiosis, are formed by either one or multiple groups of microbes via exchanges of materials, energy, and information [39]. Such microbial interactions can act as a type of selection force to deterministically govern the community assembly and thus regulate microbial community structure [40, 41, 42]. Microbial co‐occurrence networks can mechanistically unravel such complex ecological relationships and offer insights regarding the community structure and stability [39, 43, 44]. Recent awareness of this has led to a surge of studies exploring variations in the properties of microbe–microbe association networks under different habitats or stresses [28, 34, 38, 45, 46]. Although co‐occurrence network analysis may not always indicate true interactions [47, 48], it can help to understand microbiome complexity and its responses to climate change [49, 50, 51]. For example, Wang et al. [26] investigated three habitats spanning 3700 km in northern China and showed that higher precipitation increased microbial network complexity. A recent study conducted at a global change experimental facility in Germany also showed that future climate conditions, including altered precipitation, increase the bacterial–fungal network complexity [52]. Importantly, the microbial ecological network complexity has been demonstrated to be an important driver of soil multifunctionality, and may even determine the direction and strength of diversity–function relationships [46, 53]. However, little is known regarding how the microbial network complexity responds to altered precipitation and thus participates in regulating ecosystem multifunctionality. This may weaken our capacity to predict ecosystem function variations under various future climate change scenarios, especially in dryland ecosystems that are extremely sensitive to altered precipitation regimes, such as the loess hilly region of China.

Here, we conducted a manipulation experiment to simulate in situ precipitation changes (ambient conditions and ±50% precipitation treatments) in an abandoned grassland in the loess hilly region of China. Based on historical and predicted annual precipitation in the Loess Plateau region, we used ±50% precipitation treatment to simulate future precipitation patterns (Supporting Information: Figure S1). We examined soil bacterial and fungal community structures using high‐throughput sequencing of 16S ribosomal RNA and internal transcribed spacer genes, respectively. We also obtained a data set of 17 ecosystem functions mediated by soil microbes, including nutrient provisioning, microbial growth efficiency, labile organic matter (LOM) decomposition, and recalcitrant organic matter (ROM) decomposition. We quantified the community assembly, constructed microbial co‐occurrence networks, and evaluated the network complexity and microbial interactions. We aim to answer the two following questions: (i) how altered precipitation patterns affect soil ecosystem multifunctionality; and (ii) how microbial diversity, assembly processes, and microbial network properties respond to altered precipitation and participate in regulating soil multifunctionality.

## RESULTS

### Response of soil multifunctionality to altered precipitation

**Figure 1:**
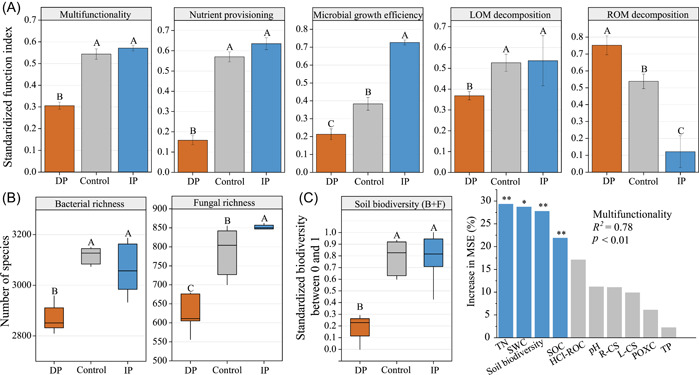
Soil multifunctionality and microbial biodiversity. (A, B) Changes in soil multifunctionality, four groups of functions, and bacterial and fungal richness. Vertical bars denote standard errors of mean values (*n* = 6). Different letters above the vertical bars indicate significant differences among treatments at *p* < 0.05 (multiple comparisons with Kruskal–Wallis tests). DP, 50% decrease in mean annual precipitation; Control, ambient precipitation; IP, 50% increase in mean annual precipitation. (C) Drivers of soil multifunctionality. Percentage increases in the MSE (mean‐squared error) of variables were used to estimate the importance of predictors, and higher MSE% values implied more important predictors. HCl‐ROC, hydrochloric acid‐resistant carbon; IP, immunoprecipitation; L‐CS, labile C structure; LOM, labile organic matter; POXC, permanganate oxidizable carbon; R‐CS, recalcitrant C structure; ROM, recalcitrant organic matter; SOC, soil organic carbon; ST, soil temperature; SWC, soil water content; TN, total nitrogen; TP, total phosphorus. Significance levels were as follows: *p < 0.05; **p < 0.01.

Decreased precipitation significantly suppressed the nutrient provisioning, microbial growth efficiency, LOM decomposition, and thus, the averaging multifunctionality by 42.6% (*p* < 0.001; Figure 1A); however, it significantly increased the ROM decomposition function by 38.9% (*p* < 0.001; Figure 1A). In contrast, increased precipitation significantly increased the microbial growth efficiency by 35.2%, but had no significant effect on the multifunctionality and other soil function groups. Analysis of C fractions further showed that decreased precipitation significantly increased the proportion of recalcitrant C structure (R‐CS) and hydrochloric acid‐resistant carbon (HCl‐ROC), whereas labile C structure (L‐CS) and permanganate oxidizable carbon (POXC) exhibited contrasting trends (Supporting Information: Figure S3). Additionally, considering the consistency of the trends among the three multifunctional indices, the averaging multifunctional index was thus used to characterize soil multifunctionality in the subsequent analysis (Supporting Information: Figure S4).

### Microbial biodiversity and its contribution to multifunctionality

Decreased precipitation resulted in markedly decreased soil bacterial and fungal richness by 7.9% and 20.9%, respectively (*p* < 0.01; Figure 1B). In contrast, increased precipitation resulted in significantly increased fungal richness by 5.3%, whereas bacterial richness remained stable. Spearman's correlation analysis indicated that the bacterial and fungal richness was significantly positively correlated with soil multifunctionality and each single function (Supporting Information: Figures S5A and S6A). Consistent results were also obtained when applying phylogenetic diversity or other multifunctionality proxies (Supporting Information: Figures S5 and S6A). Interestingly, our results showed that soil biodiversity indices that considered both bacteria and fungi together were generally more predictive of soil multifunctionality than those considering only one of the two (steeper slope; Supporting Information: Figures S5B and S6B). Random forest (RF) analysis also showed that soil biodiversity remained a significant and important predictor of ecosystem multifunctionality, even after accounting for multiple soil physicochemical properties (Figure 1C and Supporting Information: Figure S7).

### Soil microbiome assembly processes and co‐occurrence patterns

Null‐model analyses revealed that decreased precipitation significantly reduced the stochasticity of bacterial assembly but increased that of fungi (*p* < 0.01; Supporting Information: Figure S8A). Furthermore, the fungal migration (*m*‐value in the neutral community model) increased along the precipitation gradient, whereas the bacterial migration exhibited no significant changes (Supporting Information: Figure S8C). Decreased precipitation resulted in significantly decreased bacterial and fungal habitat niche breadths (*p* < 0.01; Supporting Information: Figure S8B). Conversely, increased precipitation had no significant effect on community assembly and habitat niche breadth (Supporting Information: Figure S8).

**Figure 2:**
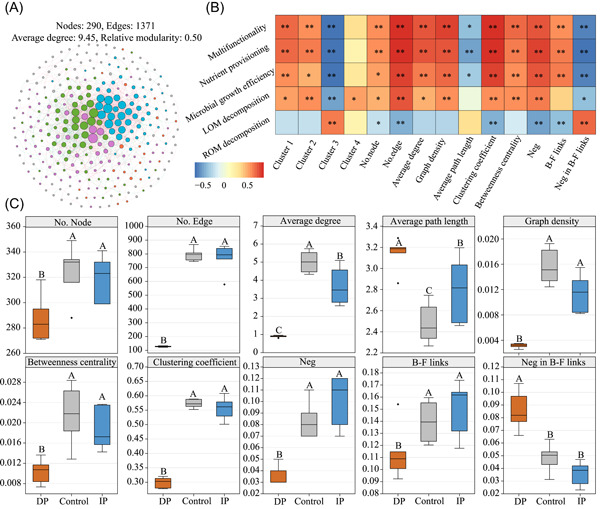
Metacommunity cross‐kingdom co‐occurrence network and relationship with soil multifunctionality. (A) Soil metacommunity cross‐kingdom co‐occurrence network. Nodes indicate individual operational taxonomic units (OTUs); the size of each node is proportional to the relative abundance of the OTUs; edges represent a significant correlation between OTUs; the width of each edge is proportional to Pearson's correlation coefficient; the colors of nodes indicate different network modules detected using the greedy modularity optimization method; red and green edges represent positive and negative relationships, respectively. (B) Spearman's correlations between soil multifunctionality and network properties. Significance levels were as follows: *p < 0.05; **p < 0.01. (C) Comparison of network topology under different treatments. Different letters indicate significant differences among treatments at *p* < 0.05 (multiple comparisons with Kruskal–Wallis tests). B–F links, the proportion of interacted links between bacteria and fungi; Control, ambient precipitation; DP, 50% decrease in mean annual precipitation; IP, 50% increase in mean annual precipitation; LOM, labile organic matter; Neg, the proportion of negative links; Neg in B–F links, the proportion of negative links between bacteria and fungi; ROM, recalcitrant organic matter.

Subsequently, we constructed a metacommunity cross‐kingdom co‐occurrence network of all samples and extracted subnetworks. We identified four dominant ecological clusters, including >80% of soil phylotypes that strongly co‐occurred within the network (Figure 2A). We found a positive correlation between the richness of soil phylotypes within two of these ecological clusters (clusters 1 and 2) and multifunctionality, whereas cluster 3 exhibited a negative correlation with multifunctionality and a positive correlation with ROM decomposition (Figure 2B and Supporting Information: Figure S9). Further analysis at the phylum level revealed that Actinobacteria, Proteobacteria, and Ascomycota were the main representative taxa in the first two clusters, whereas Acidobacteria were predominant in cluster 3 (Supporting Information: Figure S10). The topological features of the subnetwork under decreased precipitation differed significantly from those found under the control and increased precipitation conditions (Supporting Information: Figure S11). Specifically, with decreased precipitation, the number of nodes and edges, average degree, clustering coefficient, graph density, and betweenness centrality, reflecting the complexity of the network, decreased significantly (Figure 2B); however, the average path length, denoting network sparsity, increased significantly (*p* < 0.01; Figure 2B). Spearman's correlation analysis showed that all the topological parameters representing network complexity were positively correlated with soil multifunctionality, whereas the average path length was negatively correlated (Figure 2C). In addition, B–F links significantly decreased in response to decreased precipitation and were significantly positively correlated with multifunctionality, while the opposite was true for Neg in B–F links (Figure 2B,C).

Moreover, we also constructed bacterial, fungal, and cross‐kingdom networks under different treatments to estimate the network stability in response to altered precipitation. All detected networks were scale‐free and modular (Supporting Information: Table S1 and Appendix 3 of Supporting Information: Material S1). Decreased precipitation resulted in significantly decreased robustness of the bacterial, fungal, and cross‐kingdom networks while increasing their vulnerability (Supporting Information: Figures S12–14). Kolmogorov–Smirnov (K–S) tests showed that the node‐level features were significantly different between the decreased precipitation treatment and CK or increased precipitation treatment (*p* < 0.01; Supporting Information: Table S2).

### Linking microbial properties and abiotic factors to soil multifunctionality

**Figure 3:**
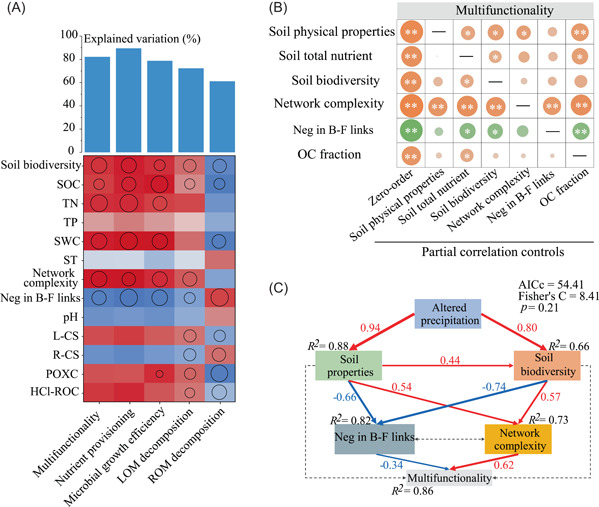
Drivers of soil multifunctionality were examined using multiple statistical approaches. (A) Contributions of multiple factors to soil multifunctionality based on Spearman's correlation and random forest model. Circle size represents the variables' importance (i.e., percentage increases of mean‐squared error calculated via random forest model). Colors represent Spearman's correlations. (B) Partial correlations between the ecosystem's multifunctionality and six types of factors. The horizontal axis shows the zero‐order (without controlling any factors) and the factors being controlled. The vertical axis shows the correlation between factors and multifunctionality. The size and color (yellow and green colors indicate positive and negative correlations, respectively) of the circles indicate the strength and sign of the correlation. Differences in circle size and color between the zero‐order and controlled factors indicate the level of dependency of the correlation between the multifunctionality and the examined factor on the controlled variable (no change in circle size and color between the controlled factor and zero‐order = no dependency; a decrease/increase in circle size and color intensity = loss /gain of correlation). Significant relationships are indicated by *p < 0.05, **p < 0.01. (C) Piecewise structural equation models showing direct and indirect effects on soil multifunctionality. Red and blue solid arrows reflect positive and negative relationships, respectively. Grey dotted arrows indicate an insignificant relationship. The width of red and blue arrows is proportional to the strength of the relationship. The numbers adjacent to the arrows are the standardized path coefficients. Soil properties represent the first component from the principal component analysis conducted for SWC, ST, pH, SOC, TN, TP, L‐CS, R‐CS, POXC and HCl‐ROC. HCl‐ROC, hydrochloric acid resistant carbon; L‐CS, labile C structure; POXC, permanganate oxidizable carbon; R‐CS, recalcitrant C structure; SOC, soil organic carbon; ST, soil temperature; SWC, soil water content; TN, total nitrogen; TP, total phosphorus.

RF analyses indicated that soil biodiversity, network complexity, and Neg in B–F links could affect the soil function (Figure 3A). Soil moisture and SOC were identified as important abiotic factors affecting soil multifunctionality. Partial correlation analysis (Figure 3B) revealed a significant and robust effect of network complexity on soil multifunctionality. After controlling for network complexity, the correlation coefficients between the other categories and soil multifunctionality decreased by 36.28%, 45.38%, 66.27%, 47.36%, and 72.42%, respectively. In contrast, the correlation coefficients between network complexity and soil multifunctionality were almost unaffected after controlling for other properties. Moreover, the soil physical properties and Neg in B–F links were found to be the two key predictors next to network complexity. Piecewise structural equation modeling (SEM) analysis (Figure 3C and Supporting Information: Figure S15) further demonstrated that network complexity and Neg‐Int directly positively and negatively regulated the soil multifunctionality, respectively. In contrast, soil biodiversity and soil properties had indirect effects through their associations with network complexity and Neg in B–F links.

## DISCUSSION

The response of soil multifunctionality to altered precipitation and its potential mechanism is a poorly explored ecological area [33]. Here, we explored the effects of ±50% precipitation variation with respect to the ambient conditions on soil multifunctionality in a semiarid grassland. The results indicate that decreased rather than increased precipitation had a significant impact on soil multifunctionality and biodiversity. Importantly, our study highlights that while taxonomic diversity is an important feature driving soil multifunctionality, it does so because diversity leads to greater microbiome complexity [34]. Our study provides insights into the mechanisms underlying the effects of global precipitation changes on soil multifunctionality.

Bacteria and fungi are predominant decomposers in soil, whose distinct growth habits are likely to respond differently to climate changes [28, 54]. Here, bacterial and fungal richness exhibited asymmetric responses to altered precipitation. The adverse effects of decreased precipitation on microbial richness might be explained by drought as a powerful environmental filter [55, 56]. For example, drought deterministically drives the microbial community assembly and reduces its diversity by reducing the water and carbon availability [57, 58]. However, this interpretation applies only to bacteria, since the decrease in fungal richness was accompanied by an increase in stochasticity. This finding, albeit somewhat surprising, is consistent with the idea that the stochastic assembly, driven by dispersal limitation, characterizes the soil fungal communities across Scotland as well as in the forests and grasslands of southern and northern China [59, 60, 61]. The fungal migration rate varied with precipitation, in stark contrast to the invariant bacterial migration rate (Supporting Information: Figure S8C). Compared to bacteria, the dispersal of fungal spores is typically restricted to shorter distances; hence, this dispersal limitation largely shapes the fungal community structure [59, 62]. This may also partly explain the positive response of fungal richness to increased precipitation, since the fungal dispersal limitation may be weakened by increased moisture [63]. An alternative, but not mutually exclusive, explanation for the increased richness in fungal communities following increased precipitation is that fungi typically predominate over bacteria due to their special physiological features (e.g., the hyphal networks that can facilitate water transport and resource acquisition) [64]. In addition, both the bacterial and fungal habitat niche breadths decreased significantly with decreasing precipitation. Generally, a microbial group with a wider niche breadth is less affected by environmental filtration and is expected to be more metabolically flexible at the community level [63, 65]. Thus, our results suggest that decreased precipitation may also increase the vulnerability of microorganisms to environmental disturbances and the risk of diversity loss by inhibiting their metabolic capacity.

The biodiversity–ecosystem function relationships have been extensively studied in recent years in both above‐ and below‐ground ecosystems [36, 66]. Here, we provide evidence that greater soil biodiversity ensured greater performance in soil functions in nutrient availability, microbial growth efficiency, and LOM decomposition. Previous large‐scale observations and microcosm experiments have also demonstrated that diversity considering more biomes facilitates improved ecosystem performance [30, 31, 34, 36]. This is mainly related to the metabolic labor division between bacteria and fungi with unique physiological characteristics leading to complementarity [34, 67]. However, the increased ROM decomposition (a less desirable function) co‐occurred with decreased diversity under precipitation reduction may be caused by the transfer of microbial substrates due to LOM depletion [68, 69]. This is supported by the analysis of organic carbon fractions and functional groups (Supporting Information: Figure S3).

In our experiments, soil multifunctionality and biodiversity (here by considering fungi and bacteria simultaneously) were relatively insensitive to increased precipitation. Historical climate features may help explain this phenomenon. For example, the prolonged semiarid climate may have shaped relatively low species richness through limited spatial carrying capacity [70]. In this case, a short‐term precipitation increase may not result in a quick significant positive effect on the local community, because the limited number of species has limited water requirements [7, 71]. Nevertheless, minor positive impacts have emerged, and over time, it is expected that the favorable soil microenvironment, via increasing moisture, may have a more positive effect on the ecosystem biodiversity and function [20, 22].

Perhaps, our most intriguing findings are in relation to the role of microbial network properties in mediating soil function under altered precipitation. First, we found a significant positive correlation between richness and multifunctionality for clusters dominated by Actinobacteria, Proteobacteria, and Ascomycota, while the opposite was true for clusters dominated by Acidobacteria. Different taxa employ different life‐history strategies, thereby directly affecting different aspects of the ecosystem function [43, 72]. For example, Actinobacteria, Proteobacteria, and Ascomycota are more competitive for the LOM under resource‐rich conditions (analogous to Y strategist) [72, 73]. In contrast, Acidobacteria preferentially invests resources in local resource acquisition (analogous to A‐strategists), especially for the depolymerization of complex organic matter in nutrient‐poor habitats. (e.g., aromatic C) [72, 74]. Second—but more importantly—we show that network complexity is a stronger and more robust factor affecting soil multifunctionality compared with biodiversity. This result aligns with previous work suggesting that more complex microbial networks contribute more to multifunctionality [30, 31, 34], as microbially derived ecological processes are not necessarily captured by the sum of coexisting individuals [75, 76]. Instead, these are the result of integrated metabolic pathways carried out by a myriad of interactions among taxa (e.g., higher resource use efficiency and metabolic regulation conferred by more tightly connected microbial members) [50, 77]. Contrary to the network complexity, the prediction of multifunctionality through species interactions has rarely been reported. Competitive species interactions have been reported to directly determine the strength and direction of the diversity–function relationship, which may lead to the collapse of bacterial communities in the presence of high diversity [53, 78]. These cases, coupled with the increased negative bacterial‐fungal associations in response to decreased precipitation in this study, raise concerns regarding the stability of ecosystem function under climate change. Competitive interactions under limited resources may lead to an overestimation of the effect of biodiversity on the ecosystem function; therefore, future studies should incorporate species interactions into ecosystem multifunctionality studies.

The interpretation of our results is hindered by the following limitations: (i) Soil organisms are widely distributed at multitrophic levels (e.g., predators with higher trophic levels [31, 36]). Although our evidence alludes that including information from multiple organisms may further improve the predictions of ecosystem function, previous studies have reported negative correlations between the ecosystem function with some predators [31]. Therefore, other unassessed trophic groups need to be further explored in manipulative experiments. (ii) As simple representations of an intricate system, co‐occurrence networks may yield spurious results [79]. However, direct inference of interactions is difficult [80]. Thus, the motivation to employ network analysis to infer interactions remains strong. In particular, network analysis is considered a valuable tool for identifying symmetric correlations between species, such as mutualism and competition [79].

## CONCLUSION

Our results reveal the response and key mediating factors of soil multifunctionality involving nutrient provisioning, microbial growth efficiency, and organic matter decomposition under altered precipitation in a semiarid grassland. We show that decreased precipitation exhibited a significant negative impact on microbial biodiversity, network complexity, and soil multifunctionality, while increased precipitation had a limited positive impact. Crucially, we found the important role of microbial network properties in regulating soil multifunctionality. Together, these results indicate that more diverse microbial communities with complicated networks may help to alleviate the adverse effects of climate change on ecosystem functioning.

## METHODS

### Site description and experimental design

Our experimental site was located at the Wuliwan watershed of the Loess Hilly region (36°51′–36°52′ N, 109°19′–109°21′ E, 1061–1371 m elevation) of the Ansai Country, Shaanxi Province, China. The site is characterized by temperate and semiarid climates, with an average annual temperature and rainfall of 8.8°C and 505 mm, respectively. The average annual potential evapotranspiration is 962.3 mm. The soil is classified as Calcaric Cambisols [81]. The region has historically experienced severe vegetation destruction and soil erosion. Since the 1970s, the Chinese government has implemented a series of vegetation restoration measures. One of the important approaches is to convert farmland into abandoned grassland for natural restoration [82]. The experimental site of this study was built on a grassland ecosystem that had been abandoned for 13 years (i.e., since 2006). Plant communities are dominated by *Astragalus melilotoides*, *Artemisia scoparia*, *Poa sphondylodes*, and *Patrinia heterophylla*.

The field experiment was initiated on a largely flat hilltop in July 2017 to investigate the effects of climate change on soil processes. The manipulation experiment used a blocked split‐plot design, in which precipitation level (−50%, −25%, ambient, +25%, +50% mean annual precipitation) was the primary factor nested with warming (whole‐year warming and unwarming) as the secondary factor. In short, the site had three experimental blocks, each including six plots. There were >5 m buffer zones between adjacent blocks. Each plot was 3 × 3 m^2^ in size and 2 m away from other plots in each block. Among six plots within one block, a control plot without any climate change factor was selected, and the remaining five plots were randomly assigned to one of the five precipitation treatments and was further divided into one warming subplot and one unwarming subplot. For this study, only the −50% precipitation (DP), ambient precipitation (Control), and +50% precipitation (IP) treatments were selected, that is, each treatment had six subsample blocks as biological replicates. For the DP treatment, multiple U‐shaped transparent plexiglass sheets with an inclination of approximately 10° were placed on a metal hanger over each plot. The transparent plexiglass covered 50% of the soil surface area, and the precipitate blocked by the U‐shaped plexiglass was collected using plastic containers. Water collected from one plot will be subsequently manually added to the nearest plot designated for IP treatment within 24 h after the end of each rainfall event. Thus, each IP subplot eventually received an additional 50% of natural precipitation.

### Soil sampling and physicochemical analyses

In July 2019, soil cores at a depth of 0–10 cm were sampled. Three soil cores (5 cm diameter) were randomly taken from each subplot and then thoroughly mixed to form a single sample. After visible roots and rocks were gently removed, the fresh soil samples were passed through a 2‐mm mesh sieve and separated into three parts. One part was air‐dried for physicochemical analyses, another part was maintained at 4°C for measuring the microbial biomass and enzyme activities, and the last part was stored at −80°C for DNA extraction. Soil pH, soil organic carbon (SOC), total nitrogen (TN), and total phosphorus (TP) were measured according to standard testing methods. We also used permanganate oxidizable carbon (POXC) and hydrochloric acid‐resistant carbon (HCl‐ROC) to characterize the labile and recalcitrant fractions of SOC, respectively [83, 84]; diffuse reflectance infrared Fourier‐transform spectroscopy analysis in the midinfrared range was used to characterize the labile and recalcitrant fractions of soil C structures. These variables were determined as detailed in Supporting Information: Material S1 (Appendix 1).

### Soil ecosystem functions

Seventeen functions associated with various aspects of soil ecosystem services were assessed and distinguished into four groups, including nutrient provisioning (soil dissolved organic C, N, and available P, and soil inorganic N [NH_4_^+^‐N and NO_3_^−^‐N]), microbial growth efficiency (microbial biomass C, N, and P, carbon use efficiency, and biomass turnover rate), LOM decomposition (extracellular enzyme activities related to sugar degradation [*β*−1,4‐glucosidase and *β*−1,4‐d‐cellobiohydrolases], chitin degradation [*β*−1,4‐*N*‐acetylglucosaminidase and leucine aminopeptidase], P mineralization [alkaline phosphatase], and ROM decomposition (lignin degradation) [peroxidases and polyphenol oxidases]. To eliminate the effects of differences in measurement scale between functions, all functional variables were standardized on a scale from 0 to 1.

To produce a quantitative multifunctionality index for each sample, we calculated each single function as well as the averaging multifunctionality index [31], which was obtained by averaging the standardized scores of all individual ecosystem functions. Additionally, to ensure that each group of ecosystem functions contributed equally to the multifunctionality, we also calculated the alternative multifunctionality index weighted by four ecosystem function groups. Finally, we calculated the principal coordinate multifunctionality index to identify the different dimensions of multifunctionality [85]. Details on the multifunctionality calculations can be found in Supporting Information: Material S1 (Appendix 2).

### Soil microbial biodiversity

A detailed description of DNA extraction and MiSeq sequencing is presented in Supporting Information: Material S1 (Appendix 3). Before calculating the soil microbial diversity, the operational taxonomic unit (OTU) tables were rarefied to a minimum number of sequences per sample (Supporting Information: Figure S2). We used OTU richness, which is a conservative and extensively used metric, as an indicator of soil microbial diversity [36]. We standardized the bacterial and fungal richness values on a scale from 0 to 1 and averaged the standardized scores to represent the diversity of both groups of taxa. In addition, phylogenetic diversity was also evaluated to ensure that the diversity metric selection did result in bias.

### Community assembly and co‐occurrence network analysis

We quantified the effect of stochastic processes shaping microbial communities using the null‐model‐based normalized stochasticity ratio (NST) [86]. Levins' niche breadth [87] was estimated using the R package “spaa” [88]. Moreover, the neutral community model was used to further assess the potential importance of stochastic processes in determining the community assembly [89]. The model evaluated the probability of a random loss of an individual in a local community being compensated by replacement through dispersal from the metacommunity by estimating the parameter *m*. NST and neutral community model were calculated using the R packages “NST” and “minpack.lm”, respectively.

To estimate the network complexity of each sample, a metacommunity interkingdom co‐occurrence network consisting of the two microbial groups for all samples was constructed, based on Pearson's correlation calculated using an approach based on random matrix theory [38]. To avoid potential spurious associations of rare OTUs from affecting reliability, data filtering was performed before correlation calculation. Only OTUs present in 9 of the 18 samples were included for calculation. We subsequently extracted the subnetworks of the individual samples and calculated their topological features, including the number of nodes and edges, average degree, clustering coefficient, average path length, graph density, and betweenness centrality using the R package “igraph.” We subsequently performed principal coordinate analysis on the topological features to obtain an index that reflects the network complexity. Note that the average path length, denoting the network sparsity, was calculated as the inverse of the variables before the calculation of the index. We also extracted the proportion of negative links (Neg) as well as the proportions of interacted links and negative links between bacteria and fungi (B–F links and Neg in B–F links, respectively). Moreover, we identified network clusters and calculated the richness of soil microorganisms within each cluster across all samples. To evaluate the differences in networks across different precipitation regimes, we constructed separate ecological networks for each treatment, while calculating the relative modularity, vulnerability, and robustness of these networks (Supporting Information: Appendix 4.2). K–S tests were performed to compare the differences in node attributes with different networks using the R package “stats” [43]. All networks were constructed using the Molecular Ecological Network Analysis Pipeline [41], and visualized using the interactive Gephi platform (https://gephi.org). Details on the construction and characterization of co‐occurrence networks can be found in Supporting Information: Material S1 (Appendix 4).

### Statistical analyses

Unless otherwise stated, all statistical analyses were conducted using the R statistical software v.4.2.0 [90]. Spearman's correlation analysis and linear regressions were used to evaluate the relationships between biodiversity and network properties with soil function, and standardized slopes were calculated. RF analysis was performed to determine the major drivers of multiple function approaches using the R package “rfPermute” [91], and the percentage increase in mean‐squared error was used to estimate the importance of the variables. Partial correlation analysis was used to determine whether and how the effect of a particular variable on soil multifunctionality depended on other variables [92], with a greater partial correlation coefficient difference between the zero‐order and controlling correlation implying a stronger effect of the factor being controlled. Partial correlation analysis was conducted using the R packages “ggm” and “psych.” Piecewise SEM was further performed to evaluate the direct and indirect relationships among the soil properties (pH, moisture, temperature, SOC, TN, TP, and OC fraction), soil biodiversity, network complexity, and average multifunctionality using the R package “piecewiseSEM” [93]. The goodness of model fit was evaluated using Akaike information criterion corrected and Fisher's *C* statistics.

## AUTHOR CONTRIBUTIONS

Xing Wang, Zekun Zhong, Xinhui Han, and Gaihe Yang conceived the project. Xing Wang, Wenjie Li, and Zekun Zhong performed the study. Xing Wang, Qi Zhang, Zhenjiao Zhang, Weichao Liu, Hanyu Liu, and Leyin Wang analyzed the data. Jing Ma, Zhenxia Li, Quanyong Liu, Chengjie Ren, and Naijia Xiao contributed ideas to the experiment. Xing Wang, Zekun Zhong, and Xinhui Han wrote and revised the manuscript.

## CONFLICT OF INTEREST STATEMENT

The authors declare no conflict of interest.

## Supporting information

Supporting information.

Supporting information.

## Data Availability

The data that support the findings of this study are available from the corresponding author upon reasonable request. The bacterial and fungal DNA sequences generated during this study are available from the National Center for Biotechnology Information's GenBank database under the project accession numbers SRP392706 (https://www.ncbi.nlm.nih.gov/sra/?term=SRP392706) and SRP392707 (https://www.ncbi.nlm.nih.gov/sra/?term=SRP392707). Supporting Information: Materials (figures, tables, scripts, graphical abstract, slides, videos, Chinese translated version and updated materials) may be found in the online DOI or iMeta Science http://www.imeta.science/.
