# Supplementary material for: Decreased soil multifunctionality is associated with altered microbial network properties under precipitation reduction in a semiarid grassland
Source: Imeta. 2023 May 2;2(2):e106. doi: 10.1002/imt2.106 (PMC10989785; doi:10.1002/imt2.106)
Supplement: Supplementary file 1 — Supporting information. [file IMT2-2-e106-s001.pdf]

Supplemental material

Decreased soil multifunctionality is associated with the altered microbial network properties under precipitation reduction in a semiarid grassland

Running title: Soil multifunctionality and microbial network

Xing Wang[1,2], Qi Zhang[1,2], Zhenjiao Zhang[1,2], Wenjie Li[1,2], Weichao Liu[1,2], Naijia Xiao[3], Hanyu Liu[1,2], Leyin Wang[1,2], Zhenxia Li[1,2], Jing Ma[1,2], Quanyong Liu[1,2], Chengjie Ren[1,2], Gaihe Yang[1,2], Zekun Zhong[4,5*], Xinhui Han[1,2*]

[1] *College of Agronomy, Northwest A&F University, Yangling, China.*

[2] *Shaanxi Engineering Research Center of Circular Agriculture, Yangling, China.*

[3] *Institute for Environmental Genomics and Department of Microbiology and Plant Biology, University of Oklahoma, Norman, USA*

[4] *Institute of Soil and Water Conservation, Northwest A&F University, Yangling, China.*

[5] *Breeding Base for State Key Lab. of Land Degradation and Ecological Restoration in northwestern China; Key Lab. of Restoration and Reconstruction of Degraded Ecosystems in northwestern China of Ministry of Education, Ningxia University, Yinchuan, China*

Correspondence

Zekun Zhong, Institute of Soil and Water Conservation, Northwest A&F University, 712100 Yangling, China. Email: zhongzekun94@gmail.com

Xinhui Han, College of Agronomy, Northwest A&F University, 712100 Yangling, China. Email: hanxinhui@nwsuaf.edu.cn

This supplementary file includes:

1. Appendix for materials and methods

Appendix 1 Method for the determination of soil organic carbon fraction

Appendix 2 Method for the determination of soil function and multifunctionality

Appendix 3 Method for DNA extraction and MiSeq sequencing

Appendix 4 Microbial co-occurrence networks construction and characterization

2. Supplementary figures

Figure S1 Changes in the annual precipitation on the Loess Plateau from 1901 to 2100. The data used for the fitting came from http://gdo-dcp.ucllnl.org/ downscaled_cMIp_projections/.

Figure S2 Rarefaction curves for the richness of bacteria and fungi. Each line represents a soil sample.

Figure S3 Comparison of organic carbon fractions under different treatments. Vertical bars denote standard errors of mean values (n = 6). Different letters above the vertical bars indicate significant differences among treatments at $P < 0.05$ (multiple comparison with Kruskal-Wallis tests). DP, 50% decrease in mean annual precipitation; Control, ambient precipitation; IP, 50% increase in mean annual precipitation.

Figure S4 Correlations between multiple functional metrics. (A-B) Linear relationships between averaging soil multifunctionality and weighted multifunctionality as well as the multifunctionality represented by the first axes from a principal coordinate analysis including the 17 functions. (C) Spearman correlations between the single soil functions and the different components (Comp) of multifunctionality represented by the multiple axes from a principal coordinate analysis. Significance levels were as follows: *$P < 0.05$, **$P < 0.01$.

Figure S5 Relationships between soil function and microbial diversity using various measures. (A) Relationships estimated by Spearman correlation. (B) Functional effects (standardized slopes) of bacteria (blue circle), fungi (black square) and their average

diversity (red triangle) index on soil function. Significance levels were as follows: *$P < 0.05$, **$P < 0.01$.

Figure S6 Relationship between single soil function and microbial diversity. (A) Spearman correlations between single soil function and microbial biodiversity. (B) Functional effects (standardized slopes) of bacteria (blue circle), fungi (black square) and their average diversity (red triangle) index on single soil functions. Significance levels were as follows: *$P < 0.05$, **$P < 0.01$.

Figure S7 Relative importance of factors for multiple soil function indices as indicated by a random forest model. Percentage increases in the MSE (mean squared error) of variables was used to estimate the importance of these predictors, and higher MSE% values implied more important predictors. Significance levels were as follows: *$P < 0.05$, **$P < 0.01$.

Figure S8 Normalized stochasticity ratio and neutral model fitting of bacterial and fungal community assembly. (A) The magnitude of normalized stochasticity ratio (NST) and mean habitat niche width of bacteria and fungi. The value of 50% as the boundary point between more deterministic (< 50%) and more stochastic (> 50%) assembly. Vertical bars denote standard errors of mean values. Different letters above the vertical bars indicate significant differences among treatments at $P < 0.05$ (multiple comparison with Kruskal-Wallis tests). (c) fit of the neutral model in microbial communities across different treatment. m indicates the estimated migration rate and R^2 indicates the fit to the neutral model. DP, 50% decrease in mean annual precipitation; Control, ambient precipitation; IP, 50% increase in mean annual precipitation.

Figure S9 Spearman correlations between single soil functions and the diversity (number of OTUs) of soil phylotypes within the main network clusters. Significance levels were as follows: *$P < 0.05$, **$P < 0.01$.

Figure S10 Total OTU richness for all samples at the phyla level within the major network clusters.

Figure S11 Principal coordinate analysis of network topologies with different treatments.

Figure S12 Bacterial co-occurrence network (A) and features (B) under different precipitation regimes. Error bar corresponds to the standard deviation of 100 repetitions of the simulation. Different letters above the vertical bars indicate significant differences among treatments at $P < 0.05$ (multiple comparison with Kruskal-Wallis tests). DP, 50% decrease in mean annual precipitation; Control, ambient precipitation; IP, 50% increase in mean annual precipitation.

Figure S13 Fungal co-occurrence network (A) and features (B) under different precipitation regimes. Error bar corresponds to the standard deviation of 100 repetitions of the simulation. Different letters above the vertical bars indicate significant differences among treatments at $P < 0.05$ (multiple comparison with Kruskal-Wallis tests). DP, 50% decrease in mean annual precipitation; Control, ambient precipitation; IP, 50% increase in mean annual precipitation.

Figure S14 Cross-kingdom co-occurrence network (A) and features (B) under different precipitation regimes. Error bar corresponds to the standard deviation of 100 repetitions of the simulation. Different letters above the vertical bars indicate significant differences among treatments at $P < 0.05$ (multiple comparison with Kruskal-Wallis tests). DP, 50% decrease in mean annual precipitation; Control, ambient precipitation; IP, 50% increase in mean annual precipitation.

Figure S15 Standardizing direct, indirect, and total effects of different factors on soil multifunctionality from structural equation model (SEM).

1. Appendix for materials and methods

Appendix 1 Method for the determination of soil organic carbon fraction

POXC was determined as described by Blair et al. [1]. Air-dried soil (containing 15 mg of C) was added to tube, and shaken with 20 ml of 333 mM potassium permanganate for 1 h at 180 rpm. The tube was then centrifuged for 10 min at 4,000 *g* and the supernatant solutions diluted by 1:250 with distilled water. The absorbance of diluted solutions was measured at 565 nm. HCl-ROC was determined as described by [2]. 1 g air-dried soil was digested with 6 M HCl at 125°C for 16 hours. The residue after the hydrolysis was separated from the supernatant by centrifugation (10 min at 6,037 *g*), and washed five times with de-deionized water to remove any chlorine residue. The remaining residue was oven-dried at 60 °C, finally the carbon content is measured by the $K_2Cr_2O_7$ oxidation method.

The labile and recalcitrant carbon functional groups of soil C were determined by the following methods as described by Rui et al. [3]. Samples were finely ground, mixed with potassium bromide (IR grade; soil: KBr ratio = 1:100, w:w), and analyzed using a Thermo Nicolet 6700 infrared spectrometer (Thermo Electron Scientific Instruments Corp). Reflectance spectra (4,000 - 400 cm^{-1}) were collected with 64 scans and a resolution of 4 cm^{-1}. We evaluated relative peak areas for the following organic functional groups: aliphatic C-H functional group of methyl and methylene groups (ALC: wavenumber 3,010 to 2,800 cm^{-1}), C-O in both poly-alcoholic and ether functional groups (PEC: wavenumber 1,170 to 1,148 cm^{-1}), aromatic C = C stretch and/or asymmetric -COO- stretch (AC: wavenumber 1,660 to 1,580 cm^{-1}), carboxylic

C=O (CC: wavenumber 1,740 to 1,700 cm^{-1}). Given that carbohydrate and aliphatic functional groups are assimilated more easily than aromatic and carboxylic compounds [4]. we categorized ALC and PEC as labile carbon structures, whereas AC and CC were categorized as recalcitrant carbon structures.

Appendix 2 Method for the determination of soil function and multifunctionality

A-2.1 Nutrient provisioning

Soil dissolved organic carbon (DOC) and nitrogen (DON) were extracted with MilliQ water and measured using a TOC analyzer (Shimadzu, Kyoto, Japan). Soil inorganic N (NH_4^+-N and NO_3^--N) and available phosphorus (AP) was extracted with 2 M KCl solution and 0.5 M $NaHCO_3$, respectively, and then were measured using a continuous flow analyser (AutoAnalyzer 3 SEAL, Bran and Luebbe Corp).

A-2.2 OM decomposition

The activities of the five extracellular enzymes (i.e., BG, CBH, NAG, LAP, and ALP) were measured fluorometrically using a 200-μM solution of substrates labelled with 4-methylumbelliferone (4-MUB) or 7-amino-4-methylcoumarin (7-AMC) [5]. To this end, we used a 96-well fluorometric microplate, which has 16 analytical replicates for each soil sample and 8 analytical replicates for blanks, negative controls, reference standards, and quench standards. Specifically, one gram of fresh soil sample was homogenized in 125 ml sodium acetate buffer (pH = 8.5) on a constant-temperature shaker (25 °C) at 180 rpm for 2 h. And then, 200 μl soil slurry and 50 μl of 200 μM fluorometric substrate were added to the microplate wells that served as the sample assay. In addition, 200 μl buffer + 50 μl of 200 μM fluorometric substrate, 200 μl buffer

+ 50 µl of 10 µM fluorescence standard solution, 200 µl soil slurry + 50 µl buffer, and 200 µl soil slurry + 50 µl of 10 µM fluorescence standard solution were also dispensed into the wells that served as the negative controls, reference standards, blanks, and quench standards, respectively. The microplates were incubated in the dark at 25 °C for 4 h. After incubation, 10 µl of 0.5 M NaOH was added to each well to terminate the reactions, and the fluorescence values were measured using a microplate fluorometer (Tecan Infinite M200, Salzburg, Austria) with the excitation and emission filters of 365 and 450 nm, respectively.

The activities of soil oxidases (PER and PO) were measured by the colorimetrical method using L-dihydroxyphenylalanine (L-DOPA) and L-DOPA + H_2O_2 as substrates, respectively [6]. Briefly, 1 g of frozen soil was homogeneously dispersed in 125 ml of sodium acetate buffer (pH = 8.5) on a constant-temperature shaker (25 °C) at 180 rpm for 2 h. Eight aliquots (150 µl each) of the prepared soil suspension and L-DOPA solution (50 µl each, 5 mM) were successively added into a clear 96-well microplate using an eight-channel micropipette. For PER, another 10 µl of 0.3% H_2O_2 were dispensed into the wells combined with the suspensions and substrates. The microplate was incubated in the dark at 25 °C for 5 h, and then 10 µl of 1 M NaOH was added to stop the reaction. Absorbance at 450 nm was measured using a microplate spectrophotometer (Tecan Infinite M200, Salzburg, Austria).

A-2.3 Microbial growth efficiency

Soil microbial biomass C (MBC), N (MBN), and P (MBP) were measured using the chloroform fumigation-extraction techniques [7−9]. We calculated the microbial

carbon use efficiency (CUE) based on the extracellular enzyme stoichiometric model [10], and further incorporated soil respiration data to calculate biomass turnover rate (BTR). Microorganisms with higher CUE can transformed more C into biomass and stabilized sequester in soils [11]. Moreover, BTR also determines the fate of C, with higher turnover rates generally promoting biomass accumulation [12]. These two microbial parameters underlie critical mechanisms in the global C cycle, especially in a climate-changing world [13,14]. We performed the calculation by the following three equations [10].

$$CUE_{C:X} = CUEmax \times [S_{C:X}] / (S_{C:X} + K_X)] \quad (1)$$

$$S_{C:X} = \{(1/EEA_{C:X}) / [(MBC/MBX) / (DOC/AX)]\} \quad (2)$$

$$\mathrm{BTR} = qCO_2 \times CUE_{C:N} / (1 - CUE_{C:N}) \quad (3)$$

Where $CUE_{C:X}$ is the CUE calculated based on C:N or C:P ratio; DOC/AX is DOC:DON or DOC:AP; K_X is the half-saturation constant with a value of 0.5; *CUEmax* represents the upper limit for microbial growth efficiency (0.6) based on thermodynamic constraints. *qCO2* (d^{-1}) is the microbial respiration rate per unit biomass. Note that although extracellular enzyme-based stoichiometric models are widely used to estimate microbial utilization efficiency, they have inherent disadvantages compared to isotope-based methods. Therefore, subsequent studies are encouraged to use isotope-based methods to accurately quantify microbial carbon use efficiency.

A-2.4 Soil respiration

To measure soil respiration (Rs), in one subplot of each plot, a thin-walled polyvinyl chloride (PVC) respiration collar (20 cm diameter and 5 cm height) was

permanently inserted approximately 3 cm into the soil to minimize the potential mechanical disturbance during the experiment establishment. Soil respiration were recorded using an IRGA infrared gas analyser (Li-8100; LiCor) monthly during May-September in 2019. While measuring soil respiration, we also measured soil temperature (ST) and water content (SWC) at the 10 cm depth using a portable temperature meter and a TDR-100 (Spectrum Technologies). Each measurement is selected to start at 9.00-11.00 am on a clear day and last for three days. We measured 18 plots in the same sequence to reduce temporal and spatial biases. In addition, we frequently clipped living plants inside the PVC collars and left the clipped plant materials on the soil within the collars. Each clipping was finished at least 1 day before the soil respiration measurement to minimize disturbance influence. We used the mean (n=15) of all soil respiration data during the measurement period to represent soil respiration during growing season in 2019.

A-2.5 Soil multifunctionality

As a human construct and multidimensional measure of ecosystem functions, multifunctionality quantifies the ability of an ecosystem to simultaneously deliver multiple ecosystem processes and services [15]. In this study, we evaluated multifunctionality through multiple approaches. (1) multiple single function [16]: multiple single functions represent the actual magnitude of multifunctionality in each metric. It is derived from the value after standardization (a common scale ranging from 0 to 1) of each directly measured metric; (2) the averaging multifunctionality index [17]: this index is obtained by averaging the standardized values of individual functions and

is the most widely used and easily available multifunctionality index; (3) weighted multifunctionality by four groups of ecosystem function [18]: this index averages ecosystem functions into four ecosystem services: nutrient provisioning, microbial growth efficiency, LOM decomposition, and ROM decomposition before calculating multifunctionality, so that each ecosystem service contributes equally to multifunctionality; (4) the principal coordinate multifunctionality index [15]: this index characterizes the different dimensions of multifunctionality by extracting the principal axes from the principal coordinate analysis of all the single functions.

Appendix 3 Method for DNA extraction and MiSeq sequencing

A-3.1 Soil DNA extraction, PCR amplification, and Illumina sequencing

Soil DNA was extracted from 0.5 g fresh soil of each sample using a FastDNA SPIN Kit for Soil (MP Biochemicals, Solon, OH, USA). The quality and concentration of DNA were determined by 1.0% agarose gel electrophoresis and a NanoDrop® ND-2000 spectrophotometer (Thermo Scientific Inc., USA) and kept at -80 °C prior to further use [19]. To profile soil bacterial communities, we amplified the V4 hypervariable region of 16S rRNA gene with the primer sets 515F and 907R [20]. For fungal communities, we amplified the ITS1 region with the primers sets ITS1-ITS2 [21]. Amplification was performed with an ABI GeneAmp® 9700 PCR thermocycler (ABI, CA, USA).

To permit multiplexing of samples, a 10-bp barcode unique to each sample was attached to the 5′ end of primers. The reaction system of bacterial PCR amplification contained 0.4 μl of each primer, 1.25 μl of template DNA (10 ng), 0.4 μl of FastPfu

polymerase (Beijing TransGen Biotech Co., Ltd, China), 2.5 μl of 10 × PCR buffer, 2.0 μl of dNTPs (2.5 mM), 5.0 μl of 5 × high enhancer, and 13.45 μl of sterile ultrapure water, in a total reaction volume of 25 μl. The PCR amplification of 16S rRNA was performed at an initial denaturation temperature of 95 °C for 3 min, followed by 27 cycles of 95 °C for 30 s, 55 °C for 30 s, and 72 °C for 45 s. A final elongation step at 72 °C was run for 10 min. The PCR reaction system of the ITS gene was also performed in a 25 μl mixture, which contained 0.5 μl of the two primers (30 μmol μl^{-1}), 1.5 μl of template DNA (10 ng), and 22.5 μl of Platinum PCR SuperMix (Invitrogen, Shanghai, China). The thermal cycling program consisted of an initial annealing temperature at 95 °C for 2 min, followed by 30 cycles with denaturation at 95 °C for 30 s, annealing at 55 °C for 30 s, extension at 72 °C for 30 s, and final extension at 72 °C for 5 min. The three replicated amplification products of bacteria and fungi in each soil sample were mixed to provide one final PCR product. Each mixed PCR product was purified using the Qiagen™ PCR purification kit (Qiagen, Valencia, California, United States) following the manufacturer's protocol, and then were eluted in sterile water. Subsequently, 2.0% agarose gel electrophoresis were used to quantified the concentration of each mixed gene (16S rRNA gene and ITS rRNA gene). Finally, the purified amplicons were pooled in equimolar and paired-end sequenced (2 × 300) on an Illumina MiSeq platform (Majorbio, Shanghai, China) according to the standard protocols.

A-3.2 Sequence processing

Raw FASTQ files of sequencing were de-multiplexed and quality-filtered using Quantitative Insights Into Microbial Ecology (QIIME, Version 1.9.1) workflow [19]. Briefly, the tags sequence, primer sequence, the sequences < 200 bp, an average quality score of < 25, and the reads containing ambiguous bases or any unresolved nucleotides were removed to obtain the high-quality sequence for subsequent analysis. Forward and reverse reads were incorporated into full-length sequences with FLASH (Fast Length Adjustment of Short reads). The above processed sequences were clustered into operational taxonomic units (OTUs) defined by 97% similarity using the UPARSE 11 pipeline [22], and chimeras were eliminated during this procedure. Subsequently, the largest number of sequences in each OTU were selected as the representative sequence to prepare the OTUs table. The ribosomal database project (RDP 11.5) classifier was used to assign obtained bacterial and fungal OTUs to taxonomic groups based on the SILVA database (version 138) and the UNITE fungal ITS database (version 8.0), respectively. The complete dataset was sent to the Sequence Read Archive (SRA) database of the NCBI under the accession numbers of PRJNA870174 for bacteria and PRJNA870219 for fungi.

A-3.3 Data processing

After quality sequencing, both bacterial diversity (959,199 high-quality sequences) and fungal diversity (1,068,716 high-quality sequences) were obtained with the 515F/907R (bacterial 16S rRNA) and ITS1-ITS2 (fungal ITS) primer sets across all soil samples, respectively. The number of bacterial sequences varied from 39,702 to 58,298 per sample, whereas the number of fungal sequences varied from 46,495 to

66,192 per sample. To minimize any bias in the distribution of taxa, the bacterial and fungal diversities of each treatment were calculated based on randomly selected sequences until the count reached saturation in the rarefaction curves. For the downstream analysis of soil bacteria, datasets were rarefied to 39,702 sequences, whereas for the downstream analysis of soil fungi, datasets were rarefied to 46,495 sequences. The OTUs were clustered at a similarity above 97% to calculate the diversity indices.

Appendix 4 Microbial co-occurrence networks construction and characterization

A-4.1 Network construction

All microbial co-occurrence networks were constructed on the basis of Pearson correlations of log-transformed OTU abundances, and then an RMT-based approach was used to automatically determine the cut-off threshold for the correlations. The RMT-based network approach has several advantages [23,24]. First, it has a sound theoretical foundation, which is based on two general laws of random matrix theory. Second, it has a mathematically defined non-arbitrary correlation cutoff, which minimizes the uncertainty in network construction and comparison. Third, it has a high tolerance for noise from non-random, system-specific features. Moreover, it provides broad applicability to ecological datasets in different formats. Therefore, we chose to build the network based on this approach to minimize the interference factors in network construction and comparison.

To avoid potential spurious associations of rare OTUs from affecting reliability, data filtering was performed prior to correlation calculation. For the metacommunity

cross-kingdom co-occurrence network, only OTUs present in 9 of the 18 samples were included for calculation. For the independent networks under each precipitation regime, only OTUs present in 4 of the 6 samples were included. The Pearson correlation coefficient was calculated based on the log-transformed relative abundances of OTUs. The correlation matrix obtained from the analysis of the RMT-based network approach was used to determine the correlation threshold for network construction. Only robust correlations with absolute values greater than or equal to the threshold were used to construct the network.

To test whether the derived empirical network was non-random and scale-free, the networks were evaluated against 100 random networks generated by randomly rewiring the links among the nodes [25]. With each randomization, the same suite of network topological properties was calculated. The means and standard deviations of these properties from the 100 randomizations were calculated and compared with those from the corresponding empirical MENs. Network randomization was performed in the Molecular Ecological Network Analysis Pipeline.

A-4.2 Network characterization

For the metacommunity cross-kingdom co-occurrence network, we extracted subnetworks by preserving the phylotypes (OTU) of individual soil samples using the induced_subgraph function in “igraph” package in R. Then, to describe and compare the topology of each subnetwork, we calculate a set of metrics: the number of nodes and edges, average degree, graph density, average path length, network diameter, clustering coefficient, and betweenness centrality. Each node represents one OTU, and

each edge represents a strong and significant correlation between two nodes; average degree refers to the average connections of each node with another unique node in the network; graph density refers to the intensity of connections among nodes; Average path length refers to the average network distance between all pairs of nodes; clustering coefficient represents the degree to which the nodes tend to cluster together; and graph density refers to the intensity of connections among nodes betweenness centrality refers to the potential influence of a particular node on the connections of other nodes. Therefore, higher numbers of nodes and edges, average degree, clustering coefficient, betweenness centrality, and graph density, and lower average path lengths suggest a more connected network, reflecting more potential complexity of soil networks. Finally, these topological features of the soil meta-community network are used to calculate the network complexity of each sample.

For the independent networks under each precipitation regime, we calculated network robustness and vulnerability to understand the effects of altered precipitation on network stability. The robustness (the resistance to node loss) of a given network is defined as proportion of the remaining species in this network after random removal to simulate species extinction. To test the effects of species removal on the remaining species, we calculated the abundance-weighted mean interaction strength (wMIS) of node *i* as

$$\mathrm{wMIS}_i = \frac{\sum_{j\neq i} b_j s_{ij}}{\sum_{j\neq i} b_j}$$

In the equation, where b_j is the relative abundance of species j and s_{ij} is the association strength between species *i* and *j*, which is measured by Pearson correlation

coefficient. The proportion of the remaining nodes was reported as the network robustness. The robustness value was defined when 50% of random nodes were removed.

The vulnerability of each node measures the relative contribution of the node to the global efficiency. The vulnerability of a network is indicated by the maximal vulnerability of nodes in the network as

$$\max\left(\frac{E - E_i}{E}\right)$$

Where E is the global efficiency and E*i* is the global efficiency after removing node *i* and its entire links. The global efficiency of a graph was calculated as the average of the efficiencies over all pairs of nodes:

$$E = \frac{1}{n(n-1)}\sum_{i \neq j}\frac{1}{d(i,j)}$$

Where d (*i,j*) is the number of edges in the shortest path between node *i* and *j*.

We also tested whether the networks were scale-free and modular. According to classification criteria [26], all networks exhibited scale-free properties (R^2 of power-law degree distribution ranging from 0.754 to 0.861). Modularity (M) measures the degree to which a network is compartmentalized into different modules, in which the nodes within a module are highly connected but have very few connections with nodes from other modules. Since the size and connectivity of different networks vary greatly, relative modularity (RM: a measure of how modular a network is compared to the average expected modularity) is more meaningful for comparing the modular structures of different networks [27]. In this study, RM was calculated as the ratio of the difference between the modularity of an empirical network and the mean of modularity from the

random networks over the mean of modularity from the random networks:

$$RM = \frac{M - \overline{Mr}}{\overline{Mr}}$$

where M is the modularity of the empirical network, and $\overline{Mr}$ is the mean of modularity from the random networks (Table S1). All the networks were highly modular based on both the modularity (> 0.4) and RM (> 0, meaning that modularities of empirical networks were larger than those of random networks).

References

1. Blair Graeme J., Lefroy Rod Db, Lisle Leanne. 1995. Soil carbon fractions based on their degree of oxidation, and the development of a carbon management index for agricultural systems. Australian journal of agricultural research **46**:1459-66. https://doi.org/10.1071/AR9951459
2. Silveira M. L., Comerford N. B., Reddy K. R., Cooper W. T., El-Rifai H. 2008. Characterization of soil organic carbon pools by acid hydrolysis. Geoderma **144**:405-14.
3. Rui Yichao, Jackson Randall D., Cotrufo M. Francesca, Sanford Gregg R., Spiesman Brian J., Deiss Leonardo, Culman Steven W., Liang Chao, Ruark Matthew D. 2022. Persistent soil carbon enhanced in Mollisols by well-managed grasslands but not annual grain or dairy forage cropping systems. Proceedings of the National Academy of Sciences **119**: e2118931119. https://doi.org/10.1073/pnas.2118931119
4. Shao Pengshuai, Lynch Laurel, Xie Hongtu, Bao Xuelian, Liang Chao. 2021. Tradeoffs among microbial life history strategies influence the fate of microbial residues in subtropical forest soils. Soil Biology and Biochemistry **153**:108112. https://doi.org/10.1016/j.soilbio.2020.108112
5. Saiya-Cork K. R., Sinsabaugh R. L., Zak D. R. 2002. The effects of long term nitrogen deposition on extracellular enzyme activity in an Acer saccharum forest soil. Soil Biology and Biochemistry **34**:1309-15. https://doi.org/10.1016/S0038-0717(02)00074-3
6. Deforest Jared L. 2009. The influence of time, storage temperature, and substrate age on potential soil enzyme activity in acidic forest soils using MUB-linked substrates and L-DOPA. Soil Biology and Biochemistry **41**:1180-6.
7. Brookes P. C., Landman Andrea, Pruden G., Jenkinson D. S. 1985. Chloroform fumigation and the release of soil nitrogen: a rapid direct extraction method to measure microbial biomass nitrogen in soil. Soil Biology and Biochemistry **17**:837-42. https://doi.org/10.1016/0038-0717（85）90144-0
8. Brookes P. C., Powlson D. S., Jenkinson D. S. 1982. Measurement of microbial biomass phosphorus in soil. Soil Biology and Biochemistry **14**:319-29. https://doi.org/10.1016/0038-0717（82）90001-3
9. Vance Eric D., Brookes Peter C., Jenkinson David S. 1987. An extraction method for measuring soil microbial biomass C. Soil Biology and Biochemistry **19**:703-7. https://doi.org/10.1016/0038-0717（87）90052-6

10. Sinsabaugh Robert L., Turner Benjamin L., Talbot Jennifer M., Waring Bonnie G., Powers Jennifer S., Kuske Cheryl R., Moorhead Daryl L., Follstad Shah Jennifer J. 2016. Stoichiometry of microbial carbon use efficiency in soils. Ecological Monographs **86**:172-89. https://doi.org/10.1890/15-2110.1
11. Domeignoz-Horta Luiz A., Pold Grace, Liu Xiao-Jun Allen, Frey Serita D., Melillo Jerry M., Deangelis Kristen M. 2020. Microbial diversity drives carbon use efficiency in a model soil. Nature communications **11**:1-10. https://doi.org/10.1038/s41467-020-17502-z
12. Hagerty Shannon B., van Groenigen Kees Jan, Allison Steven D., Hungate Bruce A., Schwartz Egbert, Koch George W., Kolka Randall K., Dijkstra Paul. 2014. Accelerated microbial turnover but constant growth efficiency with warming in soil. Nature Climate Change **4**:903-6. https://doi.org/10.1038/nclimate2361
13. Wang Chao, Qu Lingrui, Yang Liuming, Liu Dongwei, Morrissey Ember, Miao Renhui, Liu Ziping, Wang Qingkui, Fang Yunting, Bai Edith. 2021. Large-scale importance of microbial carbon use efficiency and necromass to soil organic carbon. Global Change Biology **27**:2039-48. https://doi.org/10.1111/gcb.15550
14. Ye Jian Sheng, Bradford Mark A., Dacal Marina, Maestre Fernando T., García Palacios Pablo. 2019. Increasing microbial carbon use efficiency with warming predicts soil heterotrophic respiration globally. Global Change Biology **25**:3354-64. https://doi.org/10.1111/gcb.14738
15. Manning Peter, van der Plas Fons, Soliveres Santiago, Allan Eric, Maestre Fernando T., Mace Georgina, Whittingham Mark J., Fischer Markus. 2018. Redefining ecosystem multifunctionality. Nature Ecology & Evolution **2**:427-36. https://doi.org/10.1038/s41559-017-0461-7
16. Delgado-Baquerizo Manuel, Reich Peter B., Trivedi Chanda, Eldridge David J., Abades Sebastián, Alfaro Fernando D., Bastida Felipe, Berhe Asmeret A., Cutler Nick A., Gallardo Antonio. 2020. Multiple elements of soil biodiversity drive ecosystem functions across biomes. Nature Ecology & Evolution **4**:210-20. https://doi.org/10.1038/s41559-019-1084-y
17. Maestre Fernando T., Quero José L., Gotelli Nicholas J., Escudero Adrián, Ochoa Victoria, Delgado-Baquerizo Manuel, García-Gómez Miguel, Bowker Matthew A., Soliveres Santiago, Escolar Cristina. 2012. Plant species richness and ecosystem multifunctionality in global drylands. Science **335**:214-8. https://doi.org/10.1126/science.1215442
18. Jiao Shuo, Lu Yahai, Wei Gehong. 2022. Soil multitrophic network complexity enhances the link between biodiversity and multifunctionality in agricultural systems. Global Change Biology **28**:140-53. https://doi.org/10.1111/gcb.15917
19. Caporaso J. G., Kuczynski J., Stombaugh J., Bittinger K., Bushman F. D., Costello E. K., Fierer N., Pena A. G., Goodrich J. K., Gordon J. I., Huttley G. A., Kelley S. T., Knights D., Koenig J. E., Ley R. E., Lozupone C. A., Mcdonald D., Muegge B. D., Pirrung M., Reeder J., Sevinsky J. R., Tumbaugh P. J., Walters W. A., Widmann J., Yatsunenko T., Zaneveld J., Knight R. 2010. QIIME allows analysis of high-throughput community sequencing data. Nature Methods **7**:335-6. https://doi.org/10.1038/nmeth.f.303
20. Biddle Jennifer F., Fitz-Gibbon Sorel, Schuster Stephan C., Brenchley Jean E., House Christopher H. 2008. Metagenomic signatures of the Peru Margin subseafloor biosphere show a genetically distinct environment. Proceedings of the National Academy of Sciences **105**:10583-8. https://doi.org/10.1073/pnas.0709942105
21. Mukherjee Pranab K., Chandra Jyotsna, Retuerto Mauricio, Sikaroodi Masoumeh, Brown Robert E., Jurevic Richard, Salata Robert A., Lederman Michael M., Gillevet Patrick M., Ghannoum

Mahmoud A. 2014. Oral Mycobiome Analysis of HIV-Infected Patients: Identification of Pichia as an Antagonist of Opportunistic Fungi. PLoS Pathogens **10**: e1003996. https://doi.org/10.1371/journal.ppat.1003996

22. Edgar R. C. 2013. UPARSE: highly accurate OTU sequences from microbial amplicon reads. Nature Methods **10**:996. https://doi.org/10.1038/nmeth.2604
23. Deng Ye, Jiang Yi-Huei, Yang Yunfeng, He Zhili, Luo Feng, Zhou Jizhong. 2012. Molecular ecological network analyses. BMC bioinformatics 13:1-20.
24. Yuan Mengting Maggie, Guo Xue, Wu Linwei, Zhang Y. A., Xiao Naijia, Ning Daliang, Shi Zhou, Zhou Xishu, Wu Liyou, Yang Yunfeng. 2021. Climate warming enhances microbial network complexity and stability. Nature Climate Change 11:343-8. https://doi.org/10.1038/s41558-021-00989-9
25. Maslov S., Sneppen K. 2002. Specificity and stability in topology of protein networks. Science 296:910-3. https://doi.org/10.1126/science.1065103
26. Broido Anna D., Clauset Aaron. 2019. Scale-free networks are rare. Nature Communications 10. https://doi.org/10.1038/s41467-019-08746-5
27. García-Palacios Pablo, Gross Nicolas, Gaitán Juan, Maestre Fernando T. 2018. Climate mediates the biodiversity–ecosystem stability relationship globally. Proceedings of the National Academy of Sciences 115:8400-5. https://doi.org/10.1073/pnas.1800425115

2. Supplementary figures

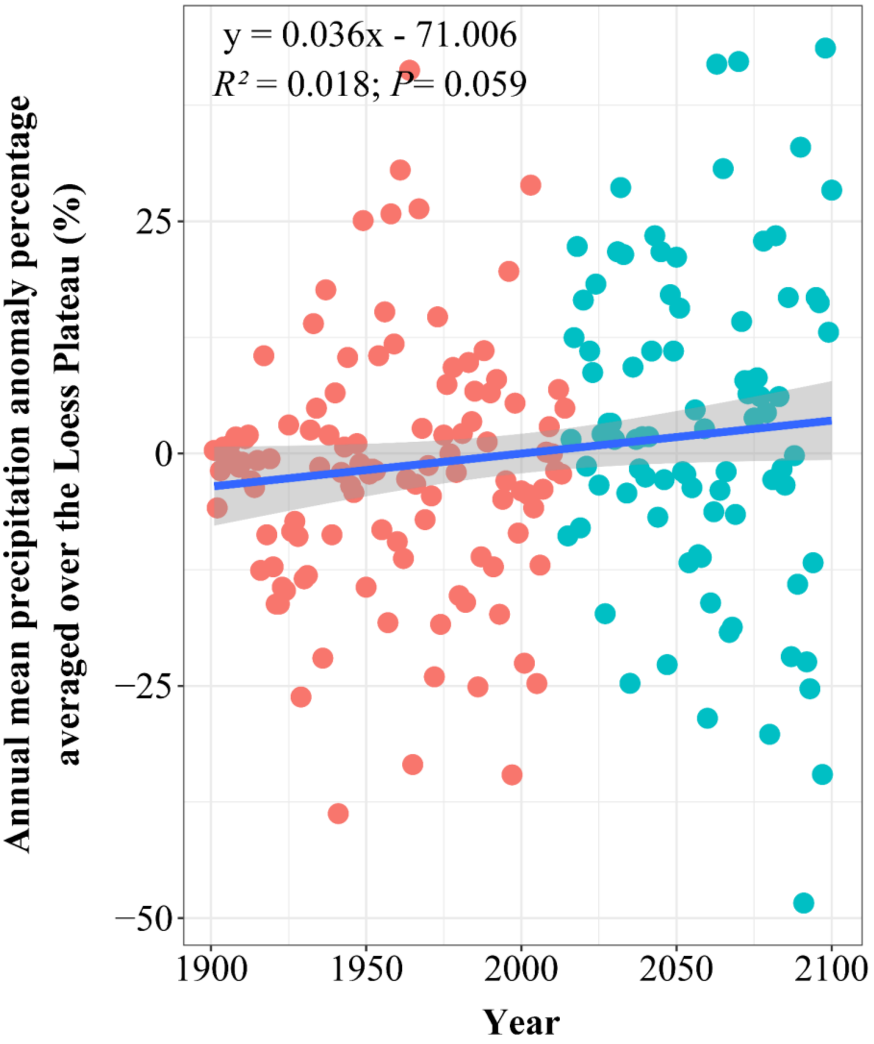

Figure S1 Changes in the annual precipitation on the Loess Plateau from 1901 to 2100. The data used for the fitting came from http://gdo-dcp.ucllnl.org/ downscaled_cMIp_projections/.

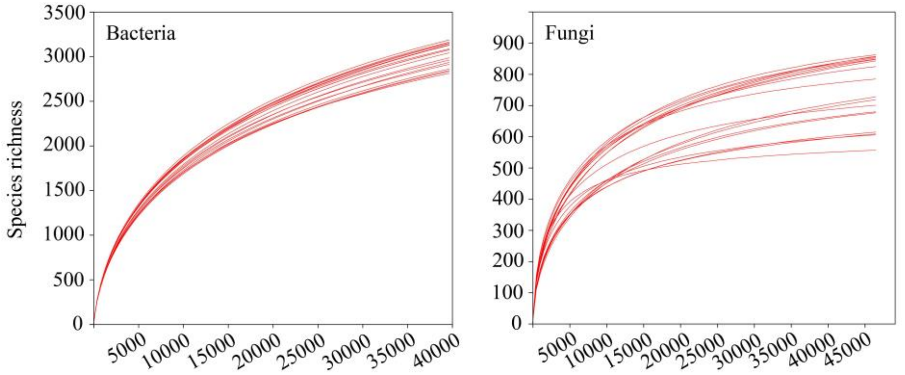

Figure S2 Rarefaction curves for the richness of bacteria and fungi. Each line represents a soil sample.

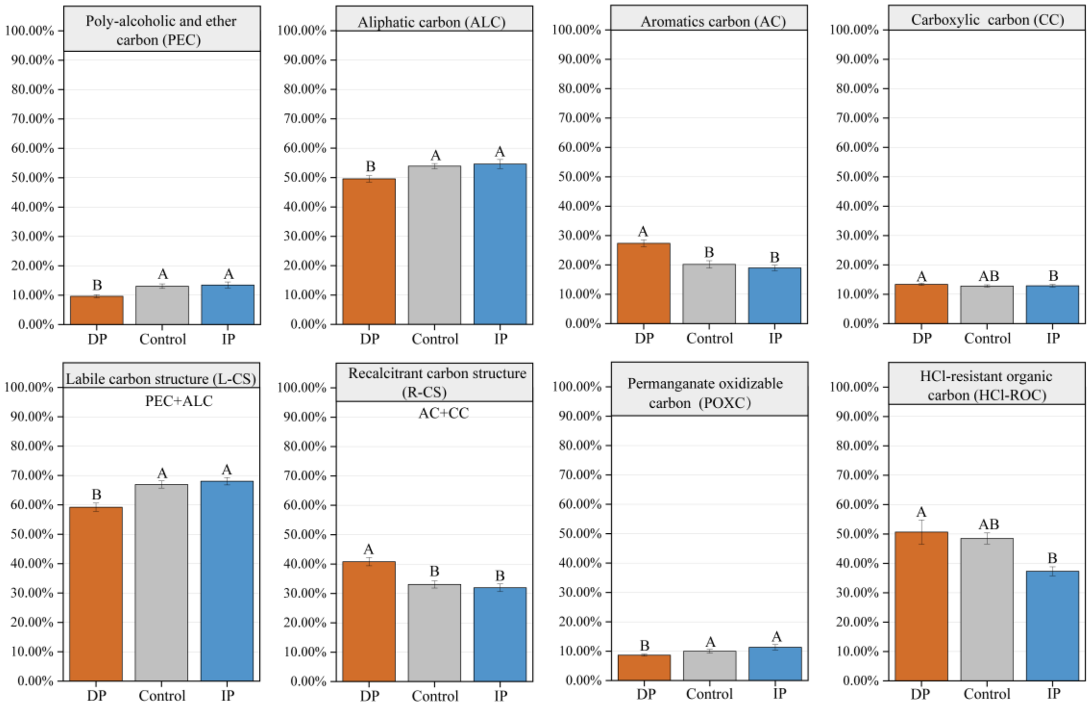

Figure S3 Comparison of organic carbon fractions under different treatments. Vertical bars denote standard errors of mean values (n = 6). Different letters above the vertical bars indicate significant differences among treatments at $P < 0.05$ (multiple comparison with Kruskal-Wallis tests). DP, 50% decrease in mean annual precipitation; Control, ambient precipitation; IP, 50% increase in mean annual precipitation.

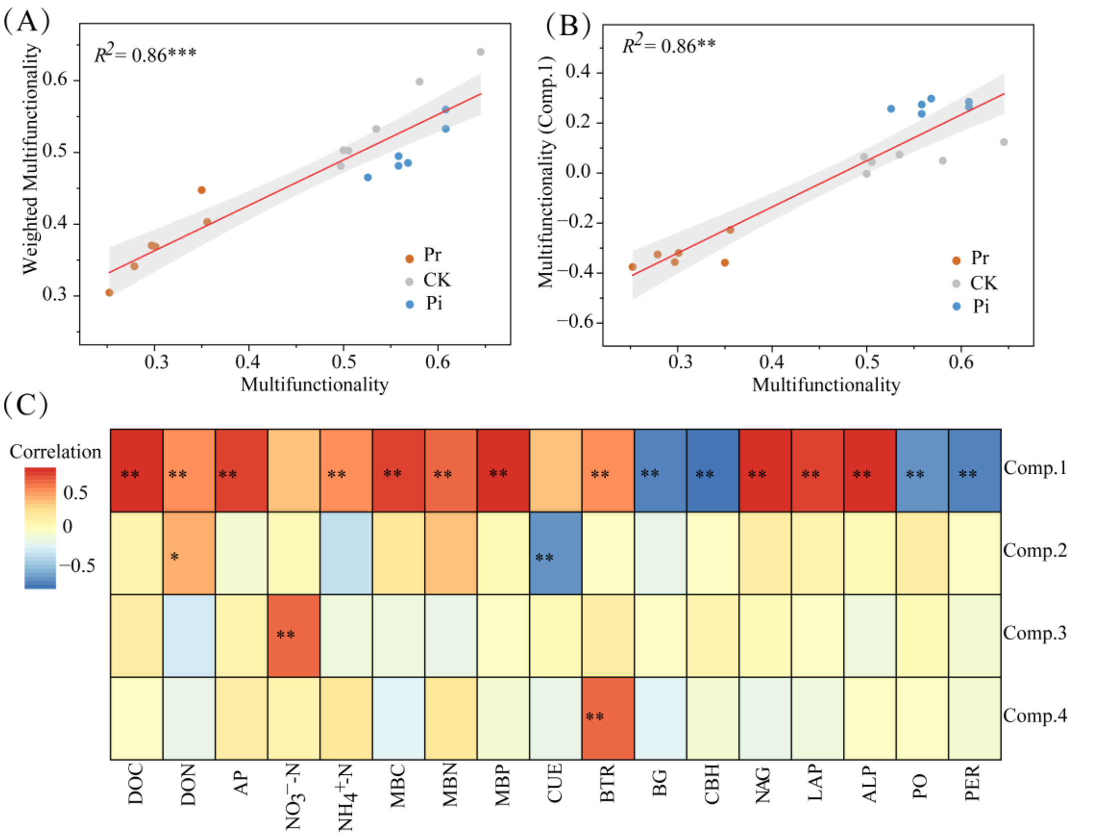

Figure S4 **Correlations between multiple functional metrics.** (A-B) Linear relationships between averaging soil multifunctionality and weighted multifunctionality as well as the multifunctionality represented by the first axes from a principal coordinate analysis including the 17 functions. (C) Spearman correlations between the single soil functions and the different components (Comp) of multifunctionality represented by the multiple axes from a principal coordinate analysis. Significance levels were as follows: *P < 0.05, **P < 0.01.

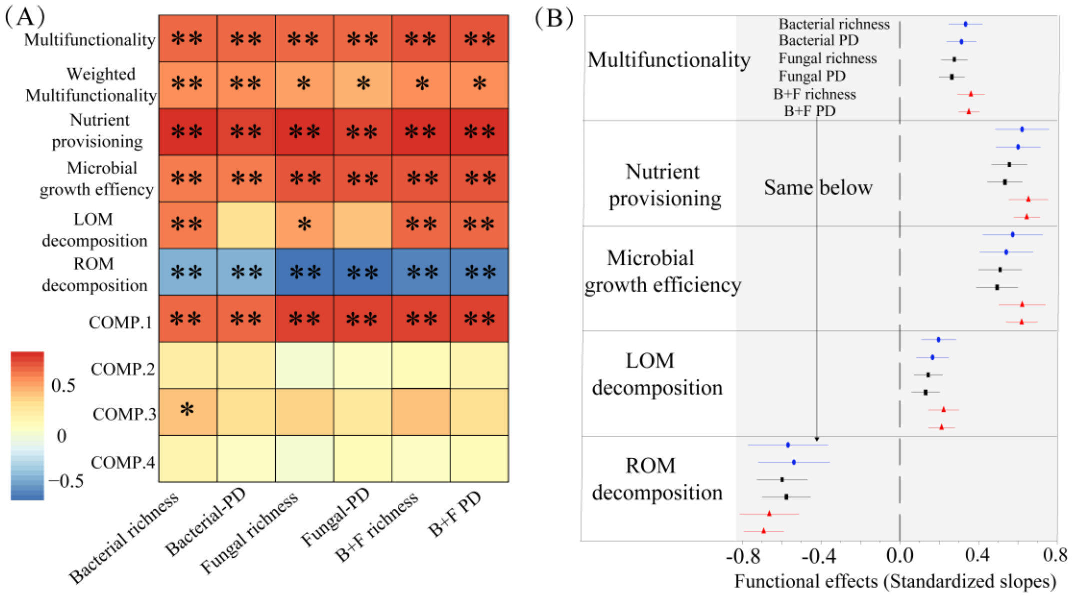

Figure S5 Relationships between soil function and microbial diversity using various measures. (A) Relationships estimated by Spearman correlation. (B) Functional effects (standardized slopes) of bacteria (blue circle), fungi (black square) and their average diversity (red triangle) index on soil function. Significance levels were as follows: **P* < 0.05, ***P* < 0.01.

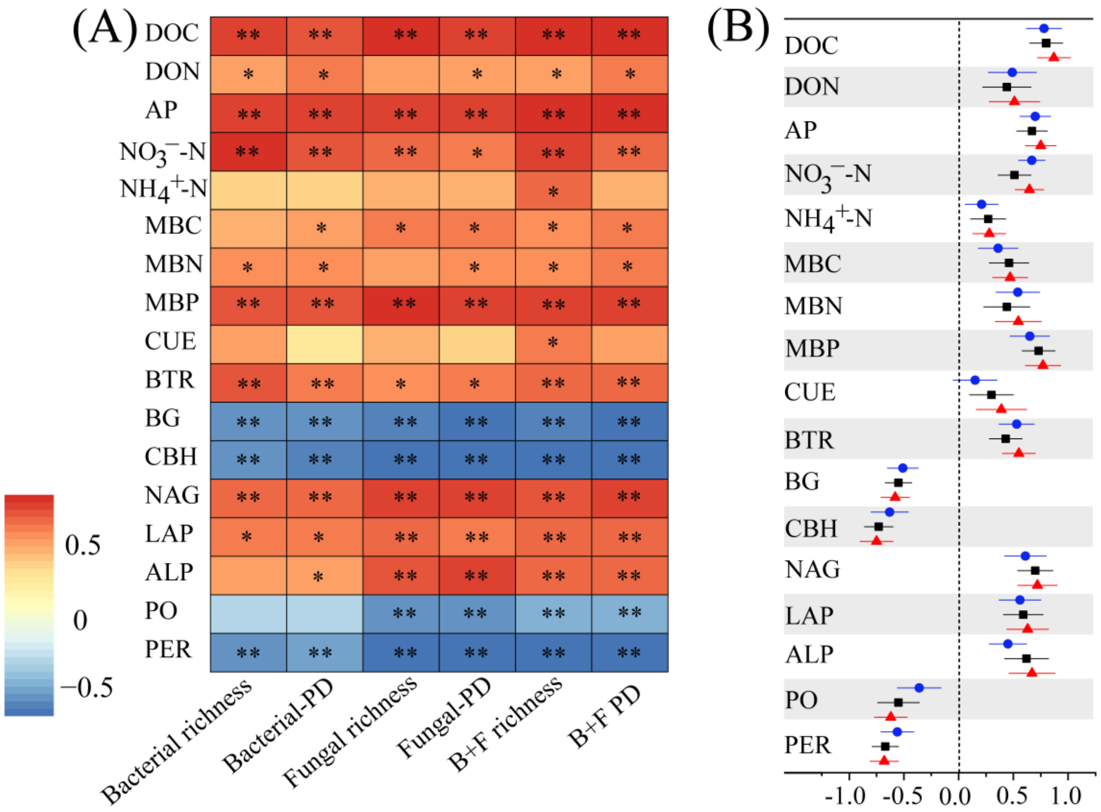

Figure S6 **Relationship between single soil function and microbial diversity.** (A) Spearman correlations between single soil function and microbial biodiversity. (B) Functional effects (standardized slopes) of bacteria (blue circle), fungi (black square) and their average diversity (red triangle) index on single soil functions. Significance levels were as follows: **P* < 0.05, ***P* < 0.01.

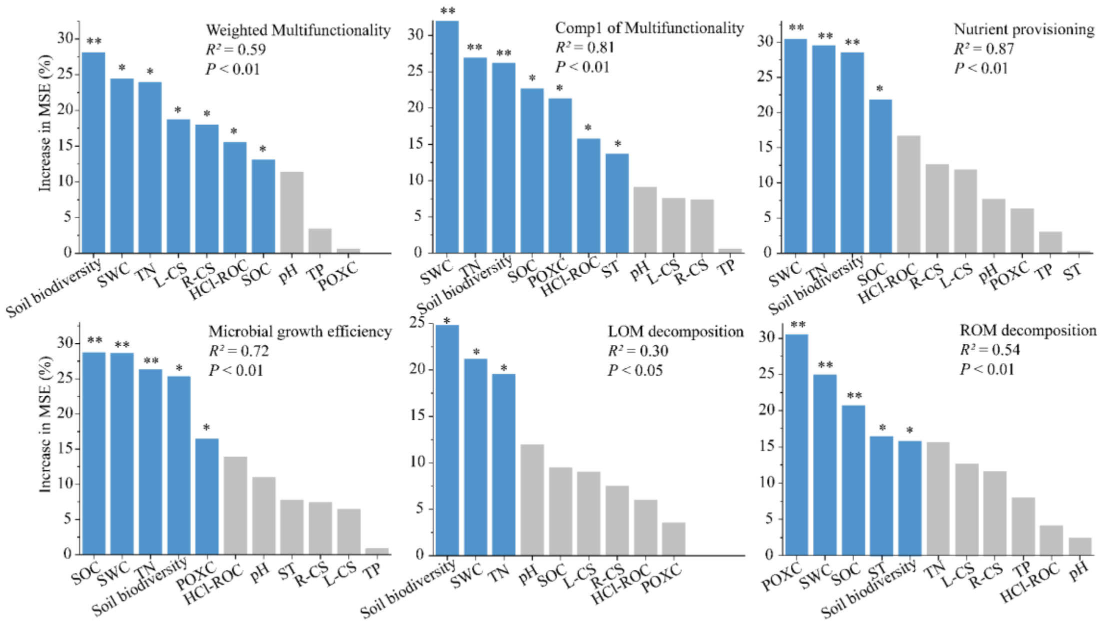

Figure S7 Relative importance of factors for multiple soil function indices as indicated by a random forest model. Percentage increases in the MSE (mean squared error) of variables was used to estimate the importance of these predictors, and higher MSE% values implied more important predictors. Significance levels were as follows: *$P < 0.05$, **$P < 0.01$.

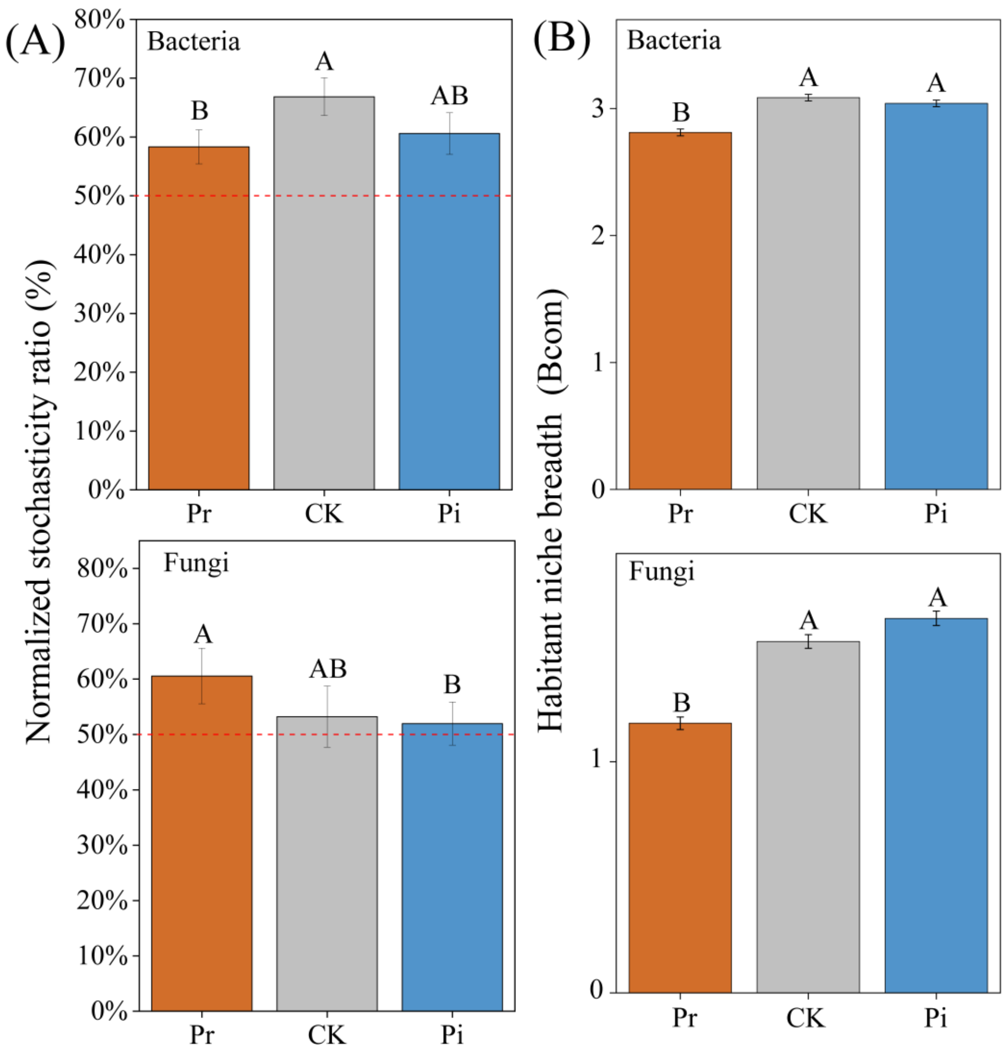

(C)

Treatments	Bacteria m	Bacteria R^2	Fungi m	Fungi R^2
DP	1.56	0.68	0.07	0.46
Control	1.63	0.65	0.15	0.45
IP	1.64	0.66	0.25	0.56

Figure S8 **Normalized stochasticity ratio (A), mean habitat niche width (B), and neutral model fitting (C) of bacterial and fungal community assembly.** The value of 50% as the boundary point between more deterministic (< 50%) and more stochastic (> 50%) assembly. Vertical bars denote standard errors of mean values. Different letters above the vertical bars indicate significant differences among treatments at $P < 0.05$ (multiple comparison with Kruskal-Wallis tests). In Panel C, m indicates the estimated migration rate and R^2 indicates the fit to the neutral model. DP, 50% decrease in mean annual precipitation; Control, ambient precipitation; IP, 50% increase in mean annual precipitation.

Single soil functions	Cluster 1	Cluster 2	Cluster 3	Cluster 4
DOC	0.78 **	0.73 **	-0.85 **	
DON		0.48 *	-0.53 *	
AP	0.57 *		-0.84 **	
NO_3^--N	0.78 **	0.69 **	-0.54 *	
NH_4^+-N			-0.56 *	
MBC	0.48 *	0.57 *	-0.76 **	
MBN	0.61 **	0.51 *	-0.74 **	0.59 **
MBP	0.57 *	0.54 *	-0.88 **	
CUE				
BTR			-0.64 **	
BG			0.81 **	
CBH	-0.52 *	-0.50 *	0.86 **	
NAG	0.70 **	0.67 **	-0.87 **	
LAP	0.69 **	0.64 **	-0.89 **	
ALP			-0.80 **	
PO			0.65 **	
PER	-0.49 *		0.73 **	

Figure S9 Spearman correlations between single soil functions and the diversity (number of OTUs) of soil phylotypes within the main network clusters. Significance levels were as follows: *$P < 0.05$, **$P < 0.01$.

Taxa at phylum level		Cluster 1	Cluster 2	Cluster 3	Cluster 4
		Richness			
Bacteria	Actinobacteria	59689	27809	10152	9729
	Proteobacteria	22916	7476	4200	5465
	Gemmatimonadetes	7867	1698	763	
	Chloroflexi	4377	619	3046	
	Acidobacteria	157	115	18369	4698
	Methylomirabilota	456		3341	789
Fungi	Ascomycota	30250	5393	7054	3328
	Basidiomycota	5126		501	2702
	Mortierellomycota	2589	2685	1167	

Figure S10 Total OTU richness for all samples at the phyla level within the major network clusters.

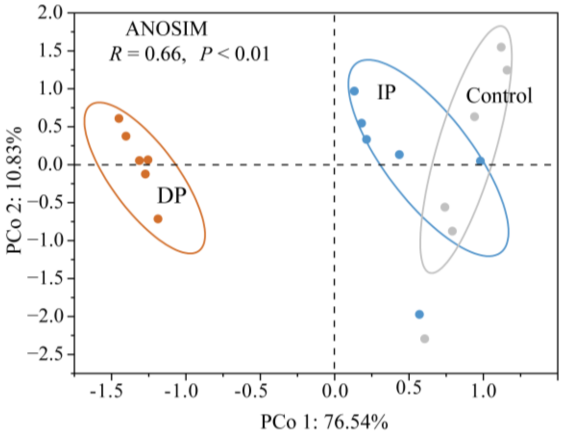

Network properties	Comp.1 98.8%	Comp.2 2.2%
No.node	0.72 **	0.62*
No.edge	0.99 **	
Average degree	0.89**	
Graph density	0.87**	
Average path length	0.64**	
Clustering coefficient	0.96**	
Betweenness centrality	0.72 **	

Figure S11 Principal coordinate analysis of network topologies with different treatments.

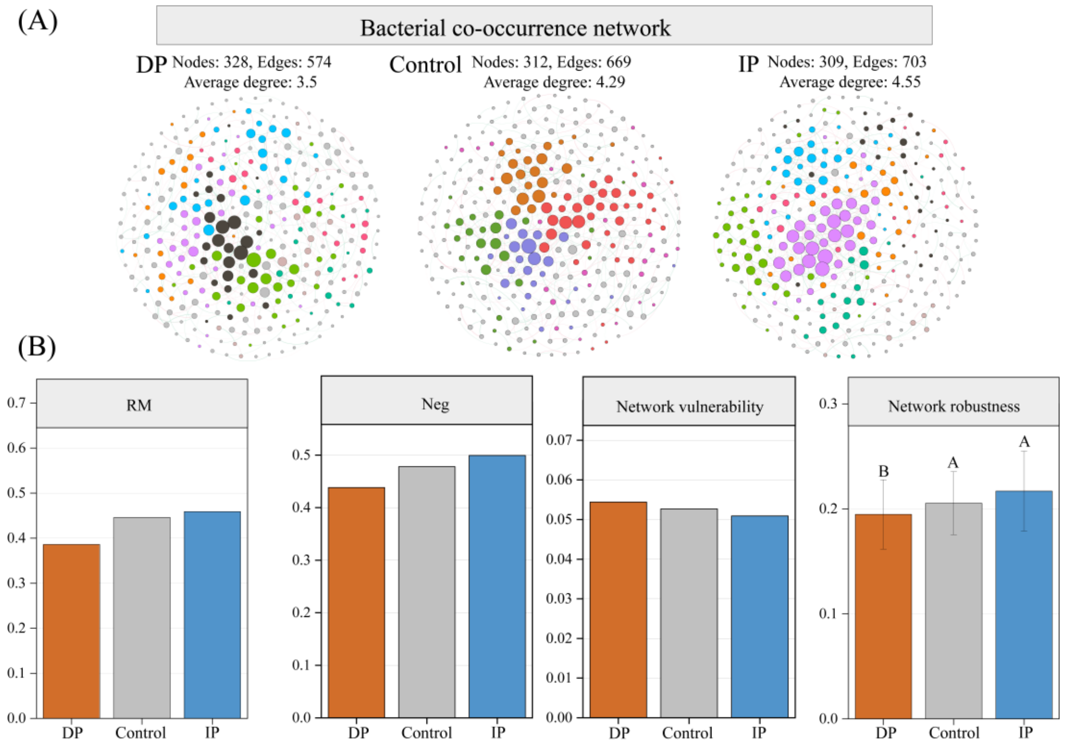

Figure S12 Bacterial co-occurrence network (A) and features (B) under different precipitation regimes. Error bar corresponds to the standard deviation of 100 repetitions of the simulation. Different letters above the vertical bars indicate significant differences among treatments at $P < 0.05$ (multiple comparison with Kruskal-Wallis tests). DP, 50% decrease in mean annual precipitation; Control, ambient precipitation; IP, 50% increase in mean annual precipitation.

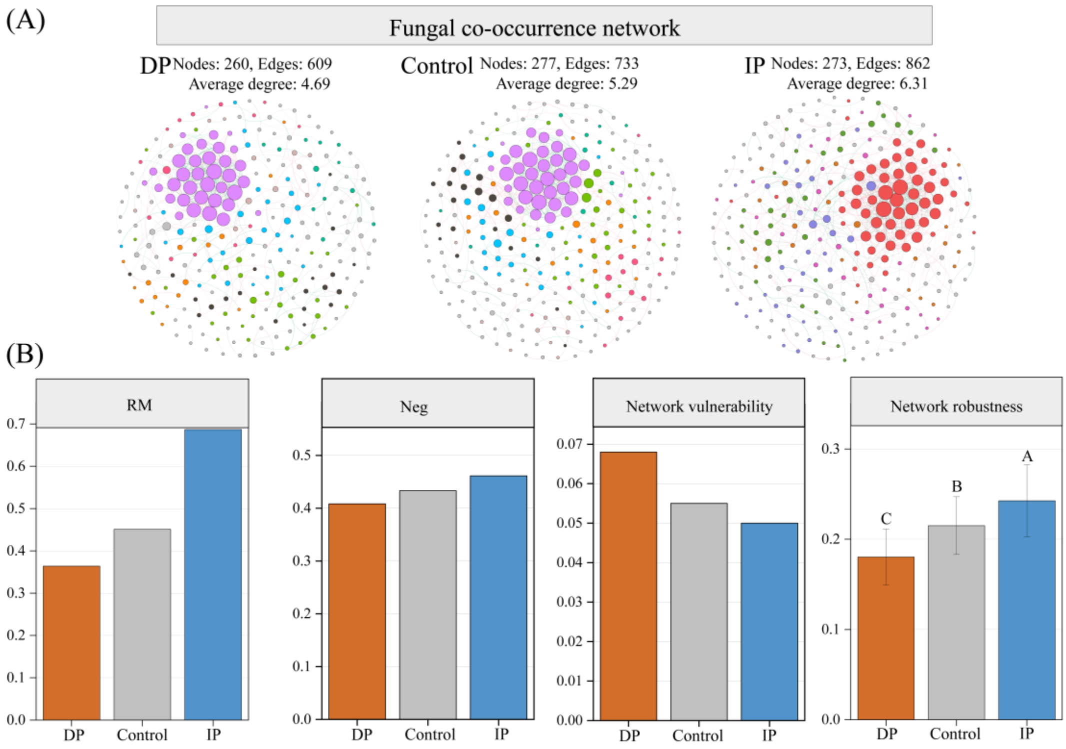

Figure S13 Fungal co-occurrence network (A) and features (B) under different precipitation regimes. Error bar corresponds to the standard deviation of 100 repetitions of the simulation. Different letters above the vertical bars indicate significant differences among treatments at $P < 0.05$ (multiple comparison with Kruskal-Wallis tests). DP, 50% decrease in mean annual precipitation; Control, ambient precipitation; IP, 50% increase in mean annual precipitation.

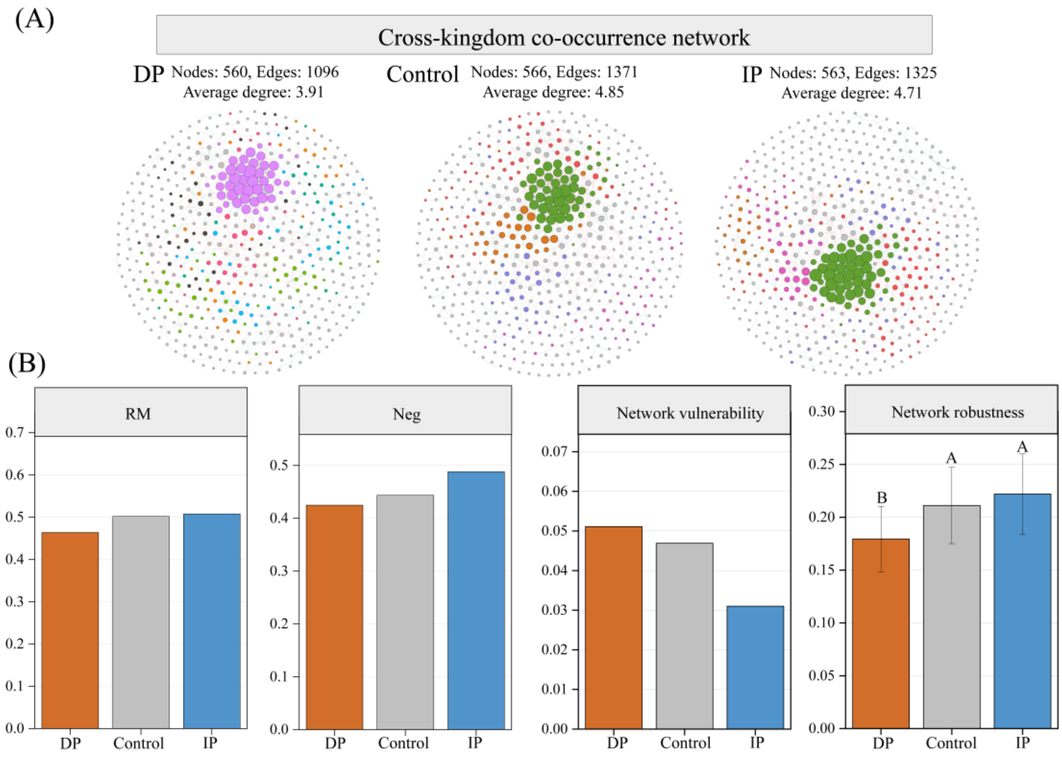

Figure S14 Cross-kingdom co-occurrence network (A) and features (B) under different precipitation regimes. Error bar corresponds to the standard deviation of 100 repetitions of the simulation. Different letters above the vertical bars indicate significant differences among treatments at $P < 0.05$ (multiple comparison with Kruskal-Wallis tests). DP, 50% decrease in mean annual precipitation; Control, ambient precipitation; IP, 50% increase in mean annual precipitation.

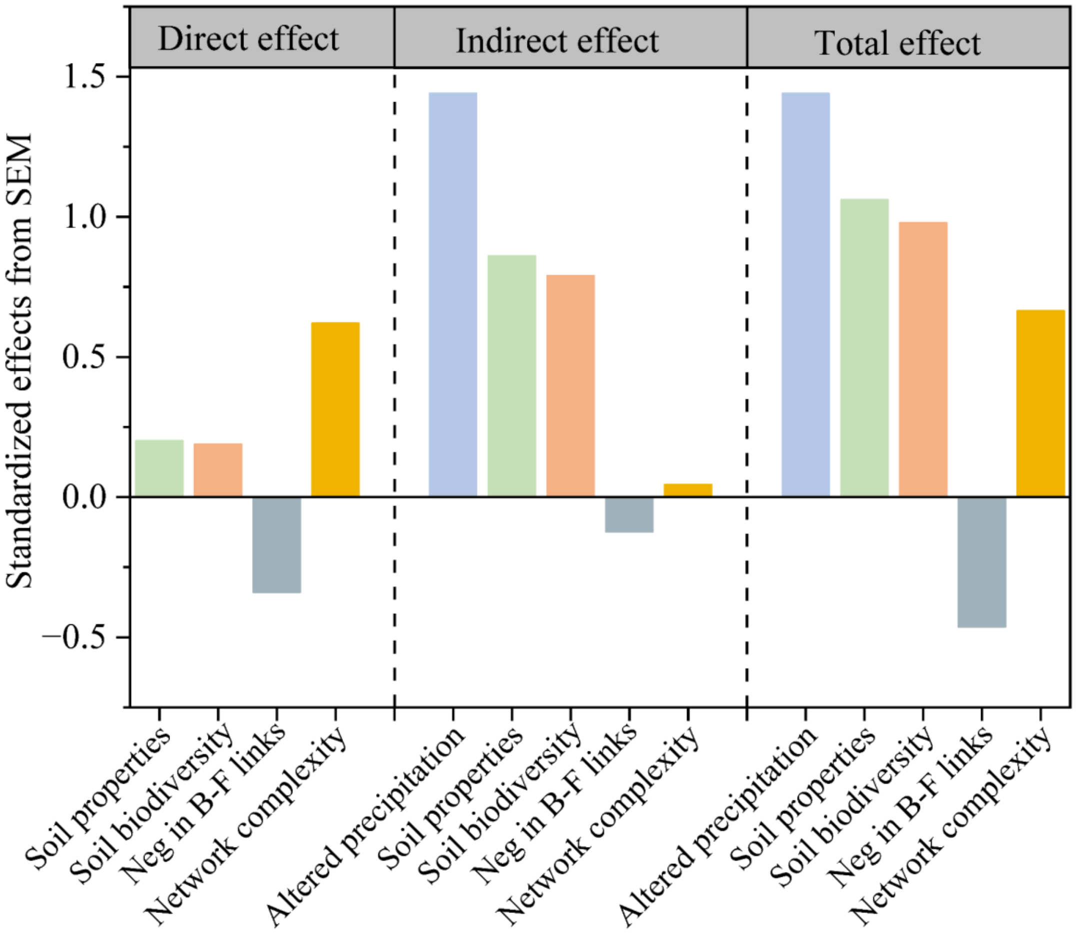

Figure S15 Standardizing direct, indirect, and total effects of different factors on soil multifunctionality from structural equation model (SEM).
